# Integrated single cell analysis of blood and cerebrospinal fluid leukocytes in multiple sclerosis

**DOI:** 10.1038/s41467-019-14118-w

**Published:** 2020-01-14

**Authors:** David Schafflick, Chenling A. Xu, Maike Hartlehnert, Michael Cole, Andreas Schulte-Mecklenbeck, Tobias Lautwein, Jolien Wolbert, Michael Heming, Sven G. Meuth, Tanja Kuhlmann, Catharina C. Gross, Heinz Wiendl, Nir Yosef, Gerd Meyer zu Horste

**Affiliations:** 1 Department of Neurology with Institute of Translational Neurology, University Hospital Münster, Münster, Germany; 2 Department of Electrical Engineering & Computer Science, Center for Computational Biology, University of California, Berkeley, CA USA; 3 Department of Physics, University of California, Berkeley, CA USA; 4 Institute of Neuropathology, University Hospital Münster, Münster, Germany; 5 Ragon Institute of MGH, MIT and Harvard, Cambridge, MA USA; 6 Chan Zuckerberg Biohub, San Francisco, CA 94158 USA

**Keywords:** Autoimmunity, Follicular T-helper cells, Biomarkers, Multiple sclerosis

## Abstract

Cerebrospinal fluid (CSF) protects the central nervous system (CNS) and analyzing CSF aids the diagnosis of CNS diseases, but our understanding of CSF leukocytes remains superficial. Here, using single cell transcriptomics, we identify a specific location-associated composition and transcriptome of CSF leukocytes. Multiple sclerosis (MS) – an autoimmune disease of the CNS – increases transcriptional diversity in blood, but increases cell type diversity in CSF including a higher abundance of cytotoxic phenotype T helper cells. An analytical approach, named cell set enrichment analysis (CSEA) identifies a cluster-independent increase of follicular (TFH) cells potentially driving the known expansion of B lineage cells in the CSF in MS. In mice, TFH cells accordingly promote B cell infiltration into the CNS and the severity of MS animal models. Immune mechanisms in MS are thus highly compartmentalized and indicate ongoing local T/B cell interaction.

## Abstract (web summary)

Here the authors provide a single-cell characterization of cerebrospinal fluid and blood of newly diagnosed multiple sclerosis (MS) patients, revealing altered composition of lymphocyte and monocyte subsets, validated by other methods including the interrogation of the TFH subset in mouse models of MS.

## Introduction

Cerebrospinal fluid (CSF) is a clear liquid that envelops and protects the central nervous system (CNS)^1^ and forms a unique local immune compartment^2^. Under healthy conditions, the noncellular fraction of CSF is mostly an ultrafiltrate of serum^3^. In contrast, CSF cells that derive exclusively from the hematopoietic lineage exhibit a tightly controlled cellular composition considerably different from the blood^4^, but the underlying mechanisms remain largely unexplored^5^. Clinically, CSF facilitates the diagnosis of inflammatory and degenerative diseases of the CNS. However, the concentration of CSF cells is ~1000-fold lower than in blood and limited volumes can be safely sampled in every patient. Technical approaches must therefore be compatible with low input and a comprehensive transcriptional characterization of single CSF cells under homeostatic conditions and in inflammatory CNS diseases is unavailable^6^.

Single-cell transcriptomics is a transformative and rapidly evolving technology that has mostly been used to redefine the heterogeneity of complex tissues from healthy rodents or humans^7,8^. Diseased tissues have also been analyzed with single-cell technologies^9^. Proponents of the technology posit that insights from single-cell transcriptomics are likely to enable precision medicine in the not-too-distant future^10^. However, outside of the field of cancer, few studies have used the technology to compare tissue samples from disease-affected vs. control donors in a clinically relevant setting. This leaves many methodological and conceptual questions unexplored.

Multiple sclerosis (MS) is a paradigmatic chronic inflammatory, demyelinating disorder of the CNS causing substantial disability^11^. This complex disease is likely of autoimmune origin, but many questions remain unanswered despite a vast amount of available literature. In fact, evidence supports the involvement of both T cells and B cells in MS, but the relative contribution of each cell type to disease etiology is unknown. On the one hand, production of immunoglobulins and expansion of B lineage cells^4^ occurs in the CSF with evidence of antigen-driven maturation^12,13^ and B-cell-depleting therapies are effective in MS^14^. On the other hand, T cells are abundant in MS lesions^15^ and T cells are affected by many established MS treatments and induce an MS-like condition named experimental autoimmune encephalomyelitis (EAE) in rodents^16^. Whether a pathological interaction of T cell and B cell subsets may occur locally in human CSF remains unknown.

Here, we apply single-cell transcriptomics to blood and CSF cells from patients with MS and controls and validate key findings. First, we identify a compartment-specific composition and transcriptome, including an unknown enrichment of myeloid dendritic cells (mDCs) in the CSF. Second, we find that MS mainly affects the cellular composition of the CSF, but the transcriptional phenotype of blood cells. We also identify an expansion of CD4^+^ T cells with a cytotoxic phenotype and late-stage B lineage cells in the CSF in MS. Third, we developed a method named cell set enrichment analysis (CSEA) to identify cluster-independent cellular changes and thereby observe an expansion of B-cell-helping T follicular helper (TFH) cells. In a reverse translational approach, we forth confirm that such TFH cells promote CNS autoimmunity and local B cell infiltration in two distinct animal models of MS. We thus demonstrate how an unbiased approach aids our understanding of a unique human immune compartment and identifies mechanisms locally driving CNS disease.

## Results

### Single-cell transcriptomics of cells in CSF and blood

**Fig. 1:**
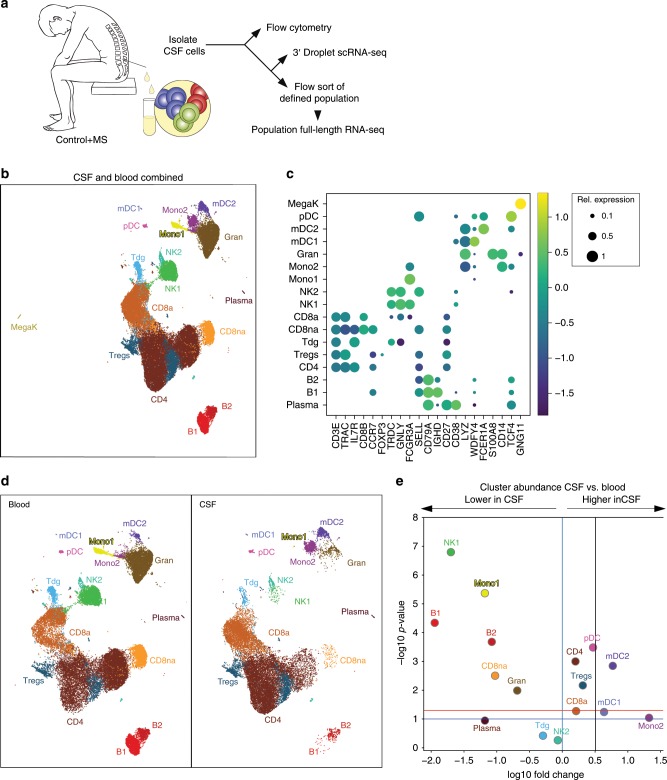
Single-cell transcriptomics reconstructs the compartment-specific leukocyte composition of CSF and blood. **a** Schematic of the study design (Methods). **b** Uniform Manifold Approximation and Projection (UMAP) plot representing 17 color-coded cell clusters identified in merged single-cell transcriptomes of blood (42,969) and CSF (22,357) cells from control (*n* = 4) and multiple sclerosis (MS; *n* = 4) patients (Methods). Cluster names were manually assigned. **c** Dotplot depicting selected marker genes in cell clusters. Dot size encodes percentage of cells expressing the gene, color encodes the average per cell gene expression level. **d** UMAP plots comparing blood (left) and CSF (right) cell clustering. Please note that the MegaK cluster is disregarded for higher resolution. **e** Volcano plot depicting differences of cluster abundance in CSF compared to blood plotting fold change (log10) against *p* value (−log10) based on beta-binomial regression (Methods). Horizontal line indicates significance threshold. Cluster key: pDC, plasmacytoid dendritic cells (DC); mDC1, myeloid DC type 1; Mono1, monocyte cluster 1 preferentially blood-derived; Mono2, monocyte cluster 2 preferentially CSF-derived; gran, granulocytes; Tdg, γδ T cells; CD8na, non-activated CD8^+^ T cells; CD8a, activated CD8^+^ T cells; Tregs, regulatory CD4^+^ T cells; CD4, CD4^+^ T cells; NK, natural killer cells; MegaK, megakaryocytes; B1/B2, B cell subsets; plasma, plasmablasts. Source data for (**c**) listing the differential expression values for all cells merged are provided in Supplementary Dataset 1. Source data for (**d**, **e**) listing the differential expression values for CSF vs. blood are provided in Supplementary Dataset 2.

We first aimed to identify the compartment-specific composition and expression of CSF cells compared to blood using an unbiased approach (Fig. 1a). We recruited patients with idiopathic intracranial hypertension (IIH) as controls and treatment-naive patients with clinically isolated syndrome (CIS) or relapsing-remitting (RR)MS (together termed MS, Methods) donating blood and CSF. Both cohorts were well matched and CSF parameters exhibited known MS-associated changes (Supplementary Fig. 1a–d, Supplementary Tables 1 and 2). Negativity for oligoclonal bands (OCB) was 18% in accordance with early MS^17^. Using microfluidics-based single-cell RNA sequencing (scRNA-seq), we obtained in total 42,969 blood single-cell transcriptomes (five control vs. five MS donors) and 22,357 corresponding CSF single-cell transcriptomes (four control vs. four MS donors). Genes detected per donor were 934.4 ± 379.1 s.e.m. in peripheral blood mononuclear cells (PBMCs) and 1,021.4 ± 374.0 s.e.m. in CSF (Supplementary Table 3). After filtering and normalization, we performed multistep clustering of the merged 65,326 blood/CSF cell dataset (Supplementary Fig. 2a). We thereby classified 61,051 single cells into 17 final cell clusters (Fig. 1b) after removal of red blood cells (RBCs) and low-quality cell clusters (Methods, Supplementary Fig. 2a). Based on the marker gene expression (Fig. 1c, Supplementary Fig. 2d, Supplementary Dataset 1; selected protein names in non-italic), we identified αβ T cells (*CD3E*, *LCK*, *TRAC*, and *TRAJ16*) subsetting into CD4^+^ T cells (*IL7R* and *CD4*), activated CD8^+^ T cells (CD8a; *CD8B* and *CCL5*), nonactivated CD8^+^ T cells (CD8na; *CD8B* and *CCR7*), regulatory T cells (*FOXP3* and *CTLA4*), and a small cluster of γδ T cells (*TRDC*). Two natural killer (NK) cell clusters (*GNLY* and *NKG7*) most likely represented the more cytotoxic and mature CD56^dim^ (NK1; *FCGR3A*/CD16 and *PRF1*), and more naive CD56^bright^ (NK2; *SELL*/CD62L and *XCL1*) subsets. Three B lineage clusters (*CD74*, *CD79A*, and *IGH* gene family) corresponded to naive B cells (B1; *CD37* and *IGHD*), activated B cells (B2; *CD27* and *IGHM*), and plasma blasts (plasma; *IGHG*, *CD38*, and *TNFRSF17*/CD269; negative for *MS4A1*/CD20 and *SDC1*/CD138). Myeloid lineage cells (*LYZ*) separated into mDC type 1 (mDC1; *WDFY4*, *XCR1*, and *BATF3*), mDC type 2 (mDC2; *FCER1A*, *CD1C*, and *CLEC10A*), and granulocytes (granulo; *S100A8* and *S100A9*). Two additional monocyte cell clusters were mostly blood-derived (Mono1; *FCGR3A*/CD16) or CSF-derived (Mono2; *CD14*). Additional clusters represented plasmacytoid dendritic cells (pDC; *TCF4/*E2-2 and *TNFRSF21/*DR6) and megakaryocytes (MegaK; *GNG11* and *CLU*). Microfluidics-based scRNA-seq thus successfully reconstructed leukocyte lineages from CSF and blood.

### CSF leukocytes exhibit a specific composition and transciptome

CSF cells have not been characterized with unbiased approaches. We therefore next analyzed the compartment-specific cell type composition identified by unbiased scRNA-seq in CSF compared to blood. As expected for CSF^4,18^, non-hematopoietic cells (e.g., neurons, glia, and ependymal cells), megakaryocytes, granulocytes, and RBCs (removed from final clustering) were absent or strongly reduced compared to blood (Fig. 1d, e, Supplementary Fig. 3a, b). We also found CD56^dim^ NK1 cells reduced among CSF cells, while the NK2 cluster was not different (Fig. 1d, e). Both the mDC1 and mDC2 clusters had a significantly higher proportion in CSF than in blood (Fig. 1d, e). Notably, mDC1 cells expressed markers indicating cross-presenting capacity (*XCR1* and *WDFY4* (ref. ^19^) Fig. 1c). Among T cells, total CD4 cells and Tregs were more abundant in the CSF, while CD8 T cell clusters were not different (Fig. 1d, e). Flow cytometry confirmed this unique composition of CSF leukocytes (Supplementary Fig. 4a–c). Cell proportions in CSF and blood did not correlate by either scRNA-seq or flow cytometry supporting an independent regulation of their cell composition. In summary, we confirmed a highly compartment-specific composition of CSF cells and identified an enrichment of mDC1 and Tregs in the CSF.

We also found a CSF-specific pattern of myeloid lineage cells. The Mono2 cluster was almost exclusively CSF-derived (Fig. 1d, Supplementary Fig. 2c) and canonical markers indicated an intermediate CD14^+^FCGR3A/CD16^int^ phenotype (Fig. 1c) as described for CSF^20^. It also expressed a unique transcriptional signature, including genes previously identified in classical (*CD9*, *CD163*, *EGR1* and *BTG2*) and in nonclassical (*C1QA*, *C1QB*, *MAF* and *CSF1R/*CD115) monocytes^21^. Notably, the CSF-derived Mono2 cluster also expressed (Supplementary Dataset 1) markers of perivascular macrophages (*LYVE1* (ref. ^22^)), microglia (*TREM2*, *TMEM119* and *GPR34* (ref. ^23^)), and CNS border-associated macrophages (*STAB1* and *CH25H* (ref. ^24,25^)) previously identified in rodents. In a systematic comparison (Methods), the Mono2 gene signatures resembled homeostatic microglia described previously^26^ (Supplementary Fig. 14a–d). We thus identified a distinct phenotype of CSF monocytes.

We next aimed to identify further compartment-specific gene expression signatures on a per cluster level (Supplementary Table 5). We focussed on genes identified independently as differentially expressed (DE) by two methods (Mann–Whitney *U* test, edgeR^27^) and supported by Bayesian model comparison in single-cell variational inference scVI ((ref. ^28^) Methods). Due to the stringency of this approach, most of such ‘triple-consistent’ genes were DE in CSF vs. blood cells in only one (18.9% of all expressed genes) or two (5.1%) clusters (Supplementary Table 5), although measures of differential expression were positively correlated especially between related clusters (Supplementary Fig. 10a), indicating coregulated gene modules in related cell types.

Genes induced in multiple (i.e., >3) CSF clusters included FGF9, previously implicated in inflammatory CNS tissue damage^29^ and metallothionein E, potentially involved in CSF metal ion homeostasis^30^. Cell cycle (e.g., *CCNC*/Cyclin-C) genes were induced in CD4^+^ T cells in line with their activated phenotype in CSF^31,32^. Genes induced in CD4^+^ T cells in the CSF were also related to lipid antigen recognition (*CD1E*), interaction with antigen-presenting cells (*CD81*, *CD83*, *CD84*, and *CD209*), and adhesion and migration (*CD99*). In fact, CSF T cells expressed a specific pattern of chemokine and integrin transcripts, including an induction of *CXCL16* and *CXCR5*, and downregulation of ITGAL/VLA4 in CSF CD4^+^ T cells and of ITGB7 in myeloid cells (Supplementary Fig. 3c, Supplementary Dataset 2). Genes consistently downregulated in CSF T cells were associated with naive cell state (*SELL*/CD62L) and cytokine responses (*IL2RG*/common γ chain). Interestingly, CD48 previously associated with CSF translocation of bacteria^33^ was upregulated in CSF T cells (Supplementary Dataset 2). In accordance, GSEA showed enrichment of pathogen response pathways in CSF-induced genes (e.g., KEGG pathways hsa05169 and hsa05168; Supplementary Dataset 3). B cell clusters (B1, B2, and plasma) showed no transcriptional changes between compartments (Supplementary Dataset 2). Genes associated with memory formation (*ID3* and *CCR2*) were induced in the CD8a cluster (Supplementary Dataset 2). Single-cell transcriptomics thus identified a location-specific transcriptional phenotype and trafficking molecule expression of CSF leukocytes.

### MS alters expression in blood and cell composition in CSF

Next, we analysed our dataset for MS-associated changes. Blood cells exhibited no significant differences in composition in MS compared to control (Supplementary Fig. 5a, b), as confirmed by flow cytometry (Supplementary Fig. 4b, c). In contrast, blood cells exhibited diverse ‘triple-consistent’ (see above and Methods) transcriptional changes (Supplementary Dataset 4), including an induction of activation markers (*ICOS*), specific cytokine receptors (*IL17RA*), and trafficking molecules (*PECAM1*/CD31, *ITGA5*/α5 integrin) in T cells (Supplementary Fig. 5c).

**Fig. 2:**
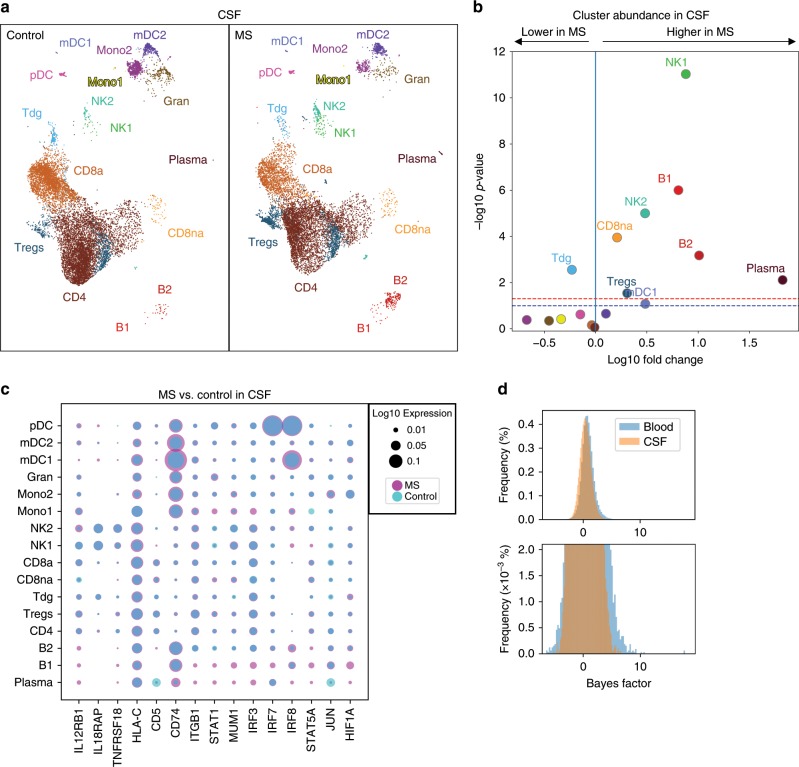
MS predominantly alters CSF cell composition and blood cell transcription. **a** Comparative UMAP plots depicting only CSF cells from control (12,705 cells, left plot) and MS (9652 cells, right plot) donors. Color coding and cluster names are as in Fig. 1. **b** Volcano plot showing differences of cluster abundance of only CSF cells in MS samples compared to controls plotted as fold change (log10) against *p* value (−log10) based on beta-binomial regression. **c** Dotplot depicting selected genes differentially expressed in at least one cluster of MS cells compared to controls in CSF. Dot size encodes percentage of cells expressing the gene. Purple indicates higher, and turquoise indicates lower expression in MS, respectively. **d** Bayes factor (BF) frequency histogram in all cluster-specific case-control differential expression analyses colored by tissue. Higher magnification in bottom panel. Only clusters with a minimum of ten cells per tissue per disease state are included. Please note that the BF is proportional to the likelihood of differential expression (i.e., higher BF indicates more likely DE)^28^. Source data for (**b**, **c**) listing the differential expression values for MS vs. controls in CSF are provided in Supplementary Dataset 5. Source data for (**d**) listing the BFs for CSF vs. blood are provided in Supplementary Dataset 2.

In contrast to blood, the cell type composition of CSF was clearly different in MS patients compared to controls (Fig. 2a, b). Using binomial regression modelling (Methods), all B lineage cell clusters (B1, B2, and plasma) significantly expanded in the CSF in MS compared to controls (Fig. 2a, b) in accordance with flow cytometry (Supplementary Fig. 4b, c) and previous studies^34–36^. Heavy chain gene expression in mature B cell clusters (B2 and plasma) was dominated by IGHG/IgG genes, although some cells expressed IGHA/IgA genes (Supplementary Fig. 6a–d). Most B lineage cells in the CSF are thus class-switched because heavy chain usage in blood evolves from *IGHD* to *IGHM* to *IGHG/IGHA* during maturation. The IGKC/κ-to-IGLC/λ ratio was at 2.75 in CSF and 1.92 in blood. Additional comparison with published signatures confirmed our B cell cluster annotation and suggested some germinal center and plasmablast phenotype cells in the plasma cluster (Supplementary Fig. 6e, f).

Among other cell lineages, both CD56^dim^ NK1 and CD56^bri^ NK2 cell clusters, and the CD8na cluster increased in the CSF in MS compared to controls (Fig. 2a, b) as confirmed by flow cytometry (Supplementary Fig. 4b, c) and in line with a previous study^37^. In addition, we identified an increase of mDC1 cells and Tregs in the CSF in MS, while γδ T cells (Tdg) were significantly decreased (Fig. 2a, b). MS thus induced complex changes of the composition of CSF leukocytes that are characterized by a simultaneous expansion of cell types with the capacity for antibody production (B1, B2, and plasma), cytotoxicity (CD8na and CD56^dim^ NK1), and with regulatory potential (Tregs and CD56^bri^ NK2).

We next tested for disease-associated ‘triple-consistent’ transcriptional changes in CSF cell clusters (Supplementary Dataset 5). In CSF T cells, we found an induction of genes associated with immune activation (*HLA-C* and *CD5*), and with interferon responses (*IL12RB1* and *IL18RAP*) and related down-stream signaling molecules (*IRF3* and *IRF8*; Fig. 2c). Specific trafficking molecules (e.g., *ITGB1*/integrin-β1) were also upregulated in MS. The CD8a cluster showed signs of increased memory formation (*ID3*). The Treg cluster showed induction of the transcription factor *STAT1* and some interferon-regulated genes (*MUM1* and *NUCB2*). The mDC2 cluster induced B-cell-related genes, (e.g., *CD79A* and *CD74*) and signs of IL-2 signaling (*STAT5A*) and a co-inhibitory molecule (*TNFRSF18*/GITR). B cell clusters did not exhibit differentially expressed genes (Supplementary Dataset 5) potentially, indicating that MS preferentially induces numerical rather than phenotypic differences in B lineage cells in the CSF. The MS-associated cellular response in CSF was thus diverse and lineage specific, and showed signs of interferon-regulated responses (Supplementary Dataset 5).

When directly comparing effects of MS between CSF and blood, we found that a greater proportion of genes was differentially expressed in blood than in CSF. For example, when performing the MS vs. control comparison, more genes (*n* = 354) were differentially expressed (DE) within the CD8a cell cluster in blood than within the same cluster in the CSF (*n* = 24). This trend toward more DE genes in blood than in MS was maintained across all cell clusters (Supplementary Datasets 4 and 5). Overall, when plotted across all clusters and genes, the Bayes factor (a measure of likelihood of differential expression that does not depend on sample size) of the MS vs. control comparison showed more extreme values in blood than in CSF (Fig. 2d). Then, we subsampled each cluster to have the same number of cells in blood and CSF and ran the Mann–Whitney *U* test and observed that the blood case-control had more significant *p* values and those *p* values were more extreme. In blood, MS thus preferentially increased transcriptional diversity, while in CSF it preferentially increased cell type diversity suggesting compartment-specific disease mechanisms.

### Cytotoxic T helper cells increase in the CSF in MS

We had tentatively handled the CD4^+^ T cell cluster as one cell type, because this population did not form clearly distinct sub-clusters (Supplementary Fig. 2a) and because many well-established T cell protein markers faired poorly on transcript level. We therefore next aimed to better characterize the CD4^+^ T cells using dedicated approaches.

**Fig. 3:**
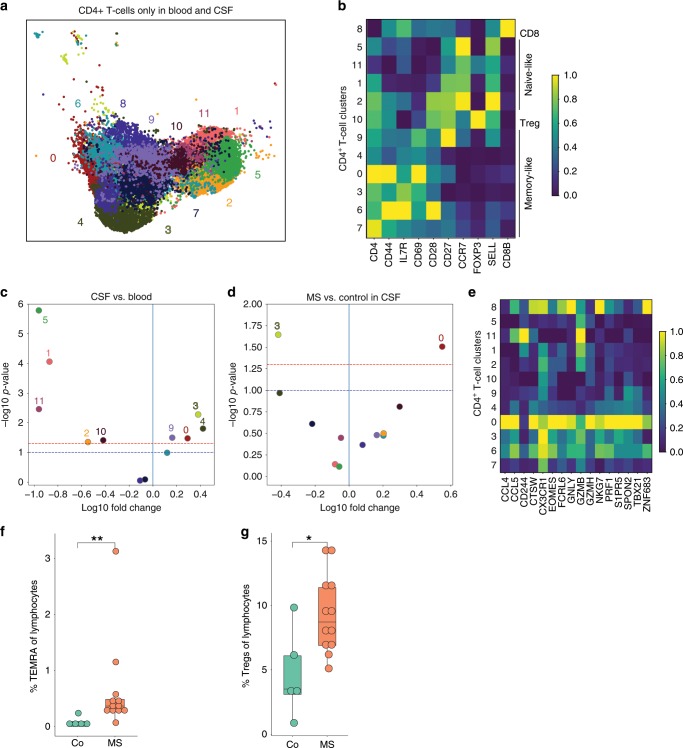
Cytotoxic-like population of CD4^+^ T cells is induced in the CSF in MS. **a** UMAP plot showing sub-clustering of all CD4^+^ T cells combined from blood (13,933 cells) and CSF (11,172 cells). Sub-clusters are numbered 0–11. **b** Heatmap depicting per cluster average expression of selected T cell subset marker genes. Expression values were normalized per gene with 0 reflecting the lowest expression and 1 reflecting the highest expression. **c** Volcano plot showing differences of CD4^+^ T cell cluster abundance in CSF compared to blood as fold change (log10) against *p* value (−log10) based on Student’s *t*-test. **d** Volcano plot showing differences of CD4^+^ T cell cluster abundance in MS compared to control within CSF based on Student’s *t*-test. **e** Heatmap showing average gene expression of selected cytotoxicity markers derived from^43^. Expression values were normalized per gene with 0 reflecting the lowest expression and 1 reflecting the highest expression. **f** The proportion of TEMRA cells (CD45RA^+^CD27^−^) among live lymphocytes in the CSF of control (co; *n* = 5) and MS (*n* = 12) patients was quantified by flow cytometry. **g** The proportion of Treg cells (CD25^high^CD127^low^) among live lymphocytes in the CSF of donors as in **f** was quantified by flow cytometry. Mann–Whitney *U* test, *p < 0.05, **p < 0.01. The lower and upper edges of the box plots represent the lower and the upper quartile, respectively, the horizontal line inside the box indicates the median, and the whiskers extend to the most extreme values within the 1.5 interquartile range of the lower/upper quartile. Source data for (**b**, **e**) listing the differential expression values for all CD4^+^ T cells are provided in Supplementary Dataset 6. Source data for (**f**, **g**) listing the TEMRA and TREGS frequencies are provided in the Source Data file.

We performed sub-clustering of the CD4^+^ T cell cluster (Fig. 3a, Supplementary Fig. 7a–c, Supplementary Dataset 6). As expected for an unsupervised clustering approach^38^, we found a minor population of CD8 T cells (*CD8B*; CD4^+^ T cell sub-cluster (CD4Tc) #8; 7.54% of all CD4^+^ T cells) remaining within the tentative CD4^+^ T cell cluster (Fig. 3a, b). The CD4^+^ T cells broadly separated into naive-like (*SELL* and *CCR7*; CD4Tc #5,11,1,2) and memory-like (*CD44*; CD4Tc #9,4,0,3,6,7) clusters based on marker gene expression (Fig. 3b). Memory cells further separated into subsets with mostly effector memory-like (*CD69*; CD4Tc #3,0,4) and central memory-like (*CD27*; CD4Tc #7,6,9) phenotype. We also identified a cluster of likely Treg identity (*FOXP3* and *CTLA4*; CD4Tc #10, Fig. 3b) located at the intersect between naive and memory cells (Fig. 3a). Notably, this cluster expressed individual markers of T cell exhaustion (*TIGIT*; Supplementary Fig. 7f)^39^ previously associated with loss of suppressive capacity of Tregs in the tumour microenvironment^40^.

We next used VISION (previously named FastProject^41^) to identify transcriptional signatures rather than individual marker genes to better interpret the CD4^+^ T cell sub-clustering. Transcriptional signatures identified a transcriptional gradient ranging from naive to memory T cell state (Supplementary Fig. 7d). This was in line with previous findings in rodents^42^, and potentially indicated that CD4^+^ T cells generally form transcriptional gradients rather than distinct sub-clusters also explaining the poor applicability of clustering approaches alone for this cell type.

We next sought to identify compartment- and disease-specific changes among CD4^+^ T cell sub-clusters. We found that several memory-type clusters (CD4Tc #3,4,0,9) were more abundant in CSF compared to blood while naive clusters (CD4Tc #1,11,2) and exhausted Tregs (CD4Tc #10) were less frequent using *t*-test based statistics (Fig. 3c; Supplementary Fig. 7e) in accordance with previous studies^31,32^. Disease-associated changes in blood were limited to a reduction of a single memory-like cluster (CD4Tc #4) in MS compared to control (Supplementary Fig. 7e). Transcriptional changes in blood and CSF did not encompass any of the key T helper (Th) cell lineage transcripts (e.g., *TBX21*, *GATA3*, and *RORC*; Supplementary Dataset 7,8). In CSF, a CD4^+^ T cell sub-cluster (2240 cells) of memory cells was significantly more abundant in MS vs. control (CD4Tc #0; Fig. 3d). This cluster expressed multiple genes associated with cytotoxic function (*GZMB*, *PRF1*, and *CCL5*) despite similar levels of CD4^+^ T cell marker genes (*CD4* and *IL7R*), low doublet probability (predicted doublet *t*-test *p* value 0.68), and absence of CD8 or NK cell markers (*CD8B* and *NKG7*; Fig. 3e) in this population. This gene signature showed considerable similarity with a recently described population of cytotoxic CD4^+^ T cells^43^ that is enriched within the CD4^+^ T cells effector memory recently activated (TEMRA) compartment. To independently confirm this, we quantified CD4^+^CD45RA^+^CD27^−^TEMRA cells and CD4^+^CD25^high^CD127^low^ Tregs by flow cytometry in the CSF of newly recruited donors (Supplementary Fig. 8). Both populations were significantly more abundant in MS than in controls (Fig. 3f, g). This indicates that cytotoxic CD4^+^ T cells and Tregs^44^ expanded in the CSF in MS.

### CSEA identifies cluster-independent transcriptional changes

Although the clustering analysis was informative about the general cell states, it was not readily able to identify a stratification of the cells into specific Th cell subsets. We therefore developed a procedure—CSEA—which reuses the GSEA test for working on ranked lists of cells rather than genes (Methods, Supplementary Fig. 7). In this procedure, the cells are first ordered by a transcriptional phenotype of interest (e.g., summed expression of genes in a pathway). The statistical test can then detect cases in which a subset of cells from one group (e.g., MS) exhibit unusually high or low values of that transcriptional phenotype compared to cells from the second group (e.g., control). We used this analysis with signature scores obtained from the VISION pipeline based on signatures obtained from databases and literature curation (Methods) to specifically analyze CD4^+^ T cells from CSF and blood.

Our CSEA testing procedure returned lists of cell sets significantly (Methods) enriched in MS and expressing a certain gene signature (Supplementary Dataset 9). The cell sets that were enriched in MS when compared to controls expressed signatures of Th cell type 1 (Th1)^45^ and TFH cells^46^ (Supplementary Fig. 9b, c). We found that the TFH signature was enriched in the CSF (*p* = 0.002) but not in the blood (*p* = 0.889). Th1 cells are significantly enriched in both blood (*p* = 0.012) and CSF (*p* = 0.0). The leading edge size reflects the number of cells driving the high enrichment score (ES). In all cases, the leading edge is small (<600 cells; Supplementary Dataset 9), indicating that a subset of cells is driving the enrichment. We also generated a random geneset that is matched to the original signature set in both number of genes and the average expression of each gene (Methods). The ES of the signature set is higher than that of the random genesets. Similar results were also obtained with more loose average expression matching (Supplementary Fig. 13). Thus, CD4^+^ T cells expressing a Th1- and TFH-like signature were enriched in MS in the CSF, but were spread across sub-clusters. Our analytical approach could therefore decouple clustering of cells from disease-state or differentiation-state enrichment of cells, providing a framework for interpreting complex scRNA-seq datasets. Interestingly, TFH cells are required for B cell maturation^47^. This lead us to hypothesize that TFH might be functionally related with the MS-specific B cell expansion in the CSF.

### The TFH subset expands in the CSF in MS and exacerbates EAE

**Fig. 4:**
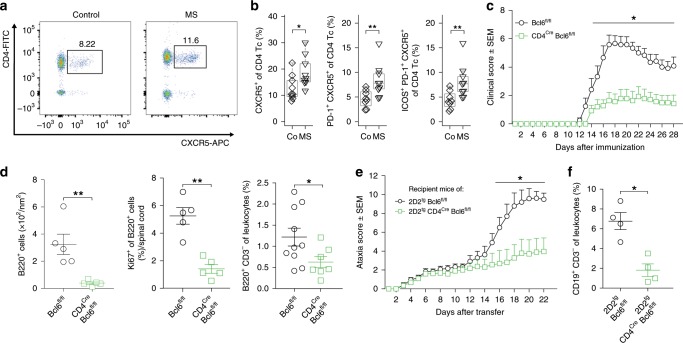
TFH cells expand in the CSF in MS and promote MS animal models. **a** Representative flow cytometry dotplot of CSF cells from a control and MS patient after gating on live CD3^+^ cells. Gating and sorting strategy is depicted in Supplementary Fig. 10a. **b** Proportion of CXCR5^+^ (left), of PD-1^+^CXCR5^+^ (middle), and of ICOS^+^PD-1^+^CXCR5^+^ (right) cells among live CD3^+^CD4^+^ T cells in CSF of control (co; *n* = 9) and MS (*n* = 9) patients quantified by flow cytometry. **c** Active EAE was induced in Bcl6^*fl/fl*^ (wildtype, circles, *n* = 6) and CD4^*Cre*^Bcl6^*fl/fl*^ (squares, *n* = 7) mice using MOG_35-55_ peptide (Methods). Mice were monitored daily for clinical EAE signs. One representative of three independent experiments is shown. **d** At day 28 after EAE induction, the density of B220^+^ leukocytes was quantified in spinal cord paraffin cross sections by histology (left). The proportion of Ki67^+^ among B220^+^ cells was quantified (middle). The proportion of CD3^−^B220^+^ cells was quantified by flow cytometry at peak of EAE (right). Gating strategy is depicted in Supplementary Fig. 16a. **e** Naive CD4 + T cells were sorted from Bcl6^fl/fl^2D2^tg^ mice (wildtype, circles, *n* = 6) and CD4^Cre^Bcl6^fl/fl^2D2^tg^ mice (squares, *n* = 8), differentiated in vitro (Methods), and intravenously injected into wild-type recipient mice at 5 × 10^6^ cells per mouse. Recipients were monitored for signs of EAE. One representative out of five independent experiments is shown. **f** At day 28 after transfer, the proportion of CD3^−^CD19^+^ leukocytes in brain and spinal cord was quantified by flow cytometry. Gating strategy is depicted in Supplementary Fig. 16b. Mann–Whitney *U* test, *p < 0.05, **p < 0.01, ***p < 0.005. The lower and upper edges of the box plots represent the lower and the upper quartile, respectively, the horizontal line inside the box indicates the median, and the whiskers extend to the most extreme values within the 1.5 interquartile range of the lower/upper quartile. **c**–**f** show mean ± s.e.m., source data listing the flow cytometry results and the clinical scores are provided in the Source Data file.

We therefore next tested whether TFH cells are in fact altered in the CSF in MS. We identified CD3^+^CD4^+^CXCR5^+^ TFH cells in the CSF by flow cytometry and found a significantly increased proportion of TFH cells in MS patients (Fig. 4a, b) in accordance with previous studies in the blood^48^ and CSF^49^. Activated PD-1^+^ and PD-1^+^ICOS^+^ TFH cells were also increased in the CSF (Fig. 4b) while the alternative CD4^+^CXCR5^−^PD-1^+^ subset^50^ was unchanged (Supplementary Fig. 12d). The percentage of PD-1^+^ TFH cells in CSF positively correlated with the proportion of CSF plasma cells quantified by flow cytometry (*r* = 0.70, *p* < 0.05). Next, we performed bulk population RNA-seq from sorted TFH cells from the CSF of MS patients (*n* = 7) vs. controls (*n* = 6) to better characterize this cell type. Surprisingly, no genes reached the significance threshold for differential expression (Supplementary Fig. 12c, Supplementary Dataset 10). This indicated that CSF-resident TFH cells increase in abundance, but do not considerably alter their phenotype in MS. We then performed GSEA and found an enrichment of gene sets (not individual genes) associated with T cell memory and pathogenicity in MS-derived TFH cells (*p* < 0.01, Bonferroni correction; Supplementary Dataset 10). Genes recurring in these enriched gene-sets (Supplementary Fig. 11) were associated with cytotoxicity (e.g., *GZMA*, *GZMK*, *CASP3*, and *CASP4*) and coinhibitory function (e.g., *KLRG1*, *TIGIT*, and *CTLA4*). Although statistically less stringent, this approach indicated that pathogenic TFH cells may expand in the CSF in MS patients.

We then tested whether TFH cells in fact promote neuro-inflammation to a functionally relevant extent using common animal models of MS. We generated mice with T-cell-restricted deficiency of Bcl6—the lineage-defining transcription factor of TFH cells^47^. Such CD4^*Cre*^Bcl6^*fl/fl*^ mice lack TFH cells and fail to mount antigen-specific B cell responses^47^, while the differentiation of other Th cell lineages (Supplementary Fig. 10a) and the composition of the peripheral immune compartment after immunization were unchanged (Supplementary Fig. 10b) as previously described^51^.

We induced active EAE using myelin oligodendrocyte glycoprotein (MOG)_35–55_ peptide in these mice and EAE severity was significantly reduced in CD4^*Cre*^Bcl6^*fl/fl*^ mice compared to Cre-negative littermates (Fig. 4c). Accordingly, the number of inflammatory lesions and infiltrated area in the spinal cord of CD4^*Cre*^Bcl6^*fl/fl*^ mice were lower than in controls (Supplementary Fig. 10c, d). We tested how the absence of TFH cells influenced B cells in the CNS and found a lower proportion of B cells (B220^+^CD3^−^) infiltrating the CNS in CD4^*Cre*^Bcl6^*fl/fl*^ mice by flow cytometry (Fig. 4d) and in the spinal cord by histology (Fig. 4d, Supplementary Fig. 10e).

Pan-T cell deficiency of Bcl6 in CD4^*Cre*^Bcl6^*fl/fl*^ mice will affect the priming phase of EAE and target both TFH cells and T follicular regulatory (TFR) cells^52^. To make a contribution of these potential confounders less likely, we next generated 2D2^*tg*^CD4^*Cre*^Bcl6^*fl/fl*^ mice expressing a T cell receptor transgene recognizing MOG^53^ to enable immunization-independent adoptive transfer EAE induction. After transfer of interleukin (IL)-17 producing myelin-reactive Th cells into wild-type hosts (Methods), 2D2^*tg*^Bcl6^*fl/fl*^ control T cells induced considerably more severe EAE than Bcl6-deficient 2D2^*tg*^CD4^*Cre*^Bcl6^*fl/fl*^ donor cells (Fig. 4e). This was despite comparable pre-transfer polarization of donor T cells (Supplementary Fig. 10f). Control recipients also showed a higher proportion of B cells in the CNS than recipients of Bcl6-deficient T cells (Fig. 4f). Taken together, our data indicated that TFH cells locally drive B cell responses in the CNS and promote MS-like autoimmunity.

## Discussion

In this study, we constructed an unbiased comparative single-cell map of blood and CSF cells. We identified a compartment-specific leukocyte transcriptome and composition including an enrichment of mDC1 and Tregs in the CSF. Monocytes in the CSF were especially distinct and partly resembled CNS border-associated macrophages. These findings emphasized the unique immune microenvironment of the CSF.

We used MS to test how a paradigmatic autoimmune disease would affect leukocytes in a compartment-specific manner. Surprisingly, we found that MS preferentially increased transcriptional diversity in blood, while it increased cell type diversity in CSF thus providing evidence for compartmentalized mechanisms driving human autoimmunity in the brain. In MS-derived CSF, we found an expansion of cytotoxic phenotype CD4^+^ T cells^43^ that could be involved in local MS pathology. We also found that clustering-based methods alone poorly capture disease-associated changes within CD4^+^ T cells and developed CSEA as a cluster-independent analytical approach to address this. This lead us to investigate TFH cells and these cells in fact expanded in MS within the CSF and promoted B cell accumulation and disease severity in MS-like animal models. Our study thereby provides a signature case for reverse translation from unbiased single-cell transcriptomics in humans to disease mechanisms in rodents.

Our unbiased approach considerably extended the available flow cytometry-based characterization of CSF leukocytes^4^. Notably, mDC1 cells abundant in the CSF-expressed markers of cross-presenting capacity (*XCR1* and *WDFY4* (ref. ^19^)), while NK2 cells in the CSF expressed the corresponding ligands (*XCL1* and *XCL2*) indicating that cell types equipped for cross-presentation and antiviral defence circulate the CSF. We also replicated the known activated/memory phenotype^32^ of CSF-resident T cells and identified a distinct pattern of adhesion molecule expression in CSF leukocytes (Fig. 3e). Such a repository of compartment-specific gene expression signatures could allow specifically targeting CSF cells in the future (e.g., CCL3 in CSF myloid cells (Supplementary Fig. 3c)). This also allowed us to provide a human confirmation beyond a previous single case study in human immunodeficiency virus (HIV)^54^ of the rodent border-associated macrophage cell phenotype^24,25^. Our findings thus lended further support to a species-independent ‘peri-CNS immune system’ involved in local autoimmunity and anti-pathogen defense.

A plethora of studies have analyzed mechanisms of neuro-inflammation^55^ albeit often equating rodent models with human MS. Unlike our CSF-focussed study, purely human transcriptional studies often relied on easily accessible PBMCs^56^. Some transcriptional studies of blood cells focussed on myelin antigen-specific T cells using predefined gene sets^56^. However, whether blood leukocytes actually constitute a suitable surrogate of disease mechanisms in MS remains unknown. A single available transcriptomic study of unsorted bulk CSF cells in MS returned signs of local B cell expansion^6^. Invasive lumbar punctures (LPs) are rarely justifiable in healthy individuals, which limits access to optimal controls in any CSF-based study. Others have used somatoform disorders with the inherent risk of misdiagnosis^18^. We chose IIH controls, because they require large volume CSF removal, are well matched with MS patients with regard to sex and age, and because basic CSF parameters and B cells are unchanged in IIH^18^. Some of the complex cellular changes we observed in MS vs. IIH may still be biased by this specific choice of controls. We also preferentially recruited untreated MS patients in (first) relapse to limit clinical complexity. The phenotype of CSF cells in remission or under MS treatments may be considerably different. The specificity of CSF cell changes in MS vs. other inflammatory CNS diseases, such as neuromyelitis optica spectrum diseases remains unknown. The transcriptomics cohort of our study is also clearly under-powered (and is not designed) to address the known intradisease heterogeneity of MS^57^. Our study, however, provides an essential reference point for future studies with this focus.

Specific Th cell lineages have long been associated with MS-like pathology in rodents, while evidence in human MS is more ambiguous^58^. Notably, blood T cells in our dataset showed some induction of Th17 cell-related signaling (*IL6R*) although most core Th17 transcriptional modules were not differentially expressed^59^. In contrast, CSF cells showed signs of Th1 cell-related signalling on the individual gene level (e.g., *IL12RB1*, *IL18RAP*, and *IRF8*), by GSEA (Supplementary Dataset 3), and when using CSEA (Supplementary Fig. 9d, e). We also found an expansion of CD4^+^ T cells with cytotoxic phenotype (CD4 Tc cluster #0) in MS vs. control patients in the CSF, but not in blood (Fig. 3). One of the marker genes in this cluster was *EOMES* (Fig. 3e) and notably *EOMES* is also a genetic risk locus for RRMS (Relapsing-remitting multiple sclerosis)^60^. Previously, EOMES^+^CD4^+^ T cells were shown to increase in the blood of patients with secondary progressive, albeit not RRMS and in late-stage EAE^61^. However, the previous study was underpowered to detect MS vs. control differences in the CSF (five total samples). Another set of studies defined cytotoxic CD4^+^ T cells by the lack of CD28 expression and these cells expanded in EAE^62^ and in the blood of RRMS patients^63^. A quantification of such cells in the CSF is unavailable. Another recent study used CytOF to quantify 35 predefined chemokine and cytokine markers in blood cells from MS patients^64^. A population of GM-CSF^+^CXCR4^+^ Th cells expanded in the peripheral blood of RRMS patients and was enriched in the CSF compared to the blood as expected for a memory population^64^. But again, no MS vs. control comparison in the CSF was provided. This highlights the unique CSF vs. blood design of our study. It remains to be tested to what extent GM-CSF^+^CXCR4^+^ Th cells represent a population with cytotoxic capacity and may overlap with our CD4 Tc cluster #0. Neither *CSF2* (encoding GM-CSF) nor *CXCR4* were detected in our dataset. In summary, although cell type definitions vary considerably between studies, CD4^+^ T cells with cytotoxic potential may locally contribute to MS pathogenesis.

We also found that TFH cells enhanced B cell enrichment in the CNS in EAE and correlated with B lineage cell abundance in the CSF. We used a genetically more rigorous approach than a previous study^65^ and our application of adoptive transfer EAE makes a contribution of TFR cells^52^ unlikely, because effector cells do not convert to Tregs in EAE^66^. We and others^49^ speculate that a pathological interaction between TFH cells and B cells in the CSF may locally drive CNS autoimmune reactions. In fact, B cell clones have long been known to, at least partially, expand in the CSF in MS^13^ together with migration from the periphery^34,35^. Previous studies support both an influx of B cells that have matured (i.e., class-switched) in the periphery and a local maturation of B cells in the CSF^67,68^. Our approach is in accordance with these studies and is unlikely to return false positives as it is unbiased and corrected for multiple-hypothesis testing. The relevance of B cells in MS is also supported by the efficacy of B-cell-depleting therapies^14^. It will be exceptionally interesting to extend our single cell study design to MS patients receiving B-cell-depleting treatments or in later disease stages. Our study provides an essential reference point for such future studies of human CSF and will likely facilitate understanding of diverse neurological diseases such as Parkinson’s and Alzheimer’s disease in the future.

## Methods

### Patient recruiting and inclusion

All sample processing had to be performed with fresh CSF cells that were processed immediately after collection, as freezing and other preservation approaches caused severe loss of transcriptional information and cell numbers in preliminary experiments in our hands. The latency from bedside to bench was <1 h in most patients. Consequently, most diagnostic information, required to diagnose MS, were unavailable at the time point of entry into our prospective study.

A total of 54 control patients and 60 MS patients were prospectively included in the study and were recruited from patients being treated in inpatient or outpatient clinics of the Department of Neurology with Institute of Translational Neurology at the University Clinic Münster. For MS patients, formal inclusion criteria for first inclusion were: (1) treatment naive patients with an episode suggestive of MS (i.e., CIS) or with RRMS diagnosed based on MAGNIMS criteria^69^, and (2) patients receiving LP for diagnostic purposes and consenting to participate. Both criteria had to be met for first inclusion. Exclusion criteria for MS patients for first inclusion were defined as: (1) questionable diagnosis of MS by clinical signs or magnetic resonance imaging (MRI) findings, and (2) secondary chronic progressive MS or primary progressive MS, (3) ongoing or previous immunomodulatory treatment. IIH patients were included, if they gave informed consent. Exclusion criteria for all patients were: (1) immunologically relevant comorbidities (e.g., rheumatologic diseases), (2) severe concomitant infectious diseases (e.g., HIV, meningitis, and encephalitis), (3) pregnancy or breastfeeding, (4) younger than 18 years, (5) mental illness impairing the ability to give informed consent, and (6) artificial blood contamination during the LP resulting in >200 RBCs/μl.

After 4 weeks of clinical follow-up from LP, we excluded MS and control patients whose diagnostic work-up revealed a diagnosis other than MS/IIH or who retrospectively otherwise did not match inclusion criteria or fulfilled exclusion criteria (Supplementary Fig. 1c). The following diagnostic tests were performed in all MS patients to exclude differential diagnoses: polymerase chain reaction (PCR) for cytomegaly virus, Ebstein-Barr virus, Human Herpes Virus-6, Herpes simplex Virus (HSV)-1, HSV-2, and Varicella-Zoster Virus in CSF. Blood tests for anti-HAV IgM, HBsAg, anti-HBc, anti-HCV, rheuma factor, Waaler-Rose Test, anti-cyclic citrullinated peptide, antinuclear antibody, anti-double strand DNA antibodies, antineutrophil cytoplasmic antibodies. CSF and serum were tested by the *Treponema pallidum* hemagglutination assay. Borrelia burgdorferi was detected in CSF and blood by ELISA. After this second exclusion step, a total of 45 treatment-naive patients with MS or CIS maintained in the study (Supplementary Tables 1, 2, Supplementary Fig. 1d). The control group consisted of 27 patients diagnosed with IIH (Supplementary Tables 1, 2).

Five to 22 months after the initial LP, we searched the local clinical database for follow-up information on all MS patients, who had not fulfilled the diagnostic criteria for clinically definite MS at the point of study entry (e.g., CIS patients; Supplementary Table 2). We then excluded additional six patients from the MS group, who were not confirmed to have developed CDMS or were lost to follow-up. After this third exclusion step, we maintained a total of 39 patients in the MS group and only the following types of patients were thus maintained in the MS group of the study:

1. Clinically defined MS diagnosed at study entry by at least two relapses and dissemination in space (DIS) in MRI after exclusion of differential diagnoses.
2. RRMS diagnosed at study entry by at least one relapse and dissemination in time identified by MRI or by OCB and DIS after exclusion of differential diagnoses^70^.
3. CIS irrespective of DIS or presence of OCB at initial sampling after exclusion of differential diagnoses, but with RRMS clinically defined by a second relapse at any time point later during follow-up.

Patients were recruited in four consecutive cohorts (Supplementary Tables 1 and 2, Supplementary Fig. 1). Cohort 1: single-cell RNA-seq of unsorted CSF cells (named scRNAseq; 6 IIH vs. 6 MS patients), cohort 2: CSF cell flow cytometry only using a general flow cytometry staining panel (named flow only; 7 IIH vs. 12 MS patients; gating in Supplementary Fig. 4), cohort 3: flow sorted CD3^+^CD4^+^CXCR5^+^ TFH cells from CSF for bulk RNA-seq (named TFH RNA-seq; 9 IIH vs. 9 MS patients), and cohort 4: CSF cell flow cytometry using a staining panel designed to quantify CD4 + TEMRA cells and Treg cells (named validation; 5 IIH vs. 12 MS patients; gating in Supplementary Fig. 4).

All patients were of Caucasian ethnicity and gave written informed consent. The study was performed in accordance with the declaration of Helsinki and was approved and supervised by the “Ethikkommission der Ärztekammer Westfalen-Lippe (ÄKWL) und der Westfälischen-Wilhelms-Universität” (Ethics Committee of the Board of Physicians of the Region Westfalen-Lippe and of the Westfälische Wilhelms-University Münster) under reference number 2015-522-f-S. R version 3.4.4 and RStudio 1.1.447 were used for the statistical analysis of clinical and human flow cytometry data.

### Sampling and flow cytometry analysis of CSF cells and blood cells

LPs were performed under sterile conditions using 20 G Sprotte Canulae (Pajunk Medical). Up to 5 ml of CSF and 3 ml of blood were collected in addition to diagnostic material. Samples were pseudonymized at collection. CSF was quickly transported to further processing and centrifuged at 300×*g* for 10 min. The supernatant was removed and CSF cells were resuspended in 5 ml of X-Vivo15 media (Lonza) and stored at 4 °C until processing. CSF flow cytometry was performed in all donors using a Navious flow cytometer (Beckman Coulter). Blood cells were incubated in VersaLyse buffer and blood and CSF cells were stained using the following anti-human antibodies (Beckman Coulter; clone names indicated): CD3 (UCHT1); CD4 (13B8.2); CD8 (B9.11); CD14 (RMO52); CD16 (3G8); CD19 (J3-119); CD25 (B1.49.9); CD27 (1A4CD27); CD45 (J.33); CD45RA (ALB11); CD56 (N901,NCAM16.2); CD127 (R34.34); CD138 (B-A38); and HLA-DR (Immu-357). For CSF scRNA-seq, CSF cells in media were centrifuged at 400 × *g* for 5 min and resuspended in 40 µl of X-Vivo15 media. A total of 5 µl of the single-cell suspension were manually counted in a Fuchs-Rosenthal chamber. The maximum of CSF cells used for input was 10,000 cells. If total available CSF cell numbers were <10,000 cells, all available cells were processed. On average 5917 cells ± 1505 s.d. (control 6167 cells ± 2614 s.d. vs. MS 5667 cells ± 1506 s.d.) CSF cells were used as input per donor. For blood scRNA-seq, blood was layered on top of Lymphoprep^TM^ (Stemcell) and gradient centrifugation was performed in accordance with manufacturer’s instructions. After centrifugation, PBMCs enriched in the interface were taken off and washed in 10 ml of X-Vivo15 media. A total of 5 µl of the single-cell suspension were manually counted in a Fuchs-Rosenthal chamber. The maximum of blood cells used for input was 10,000 cells. Details on cell numbers and gene capturing rates are provided in Supplementary Table 3.

### Generation of single-cell libraries and sequencing

Single-cell suspensions were loaded onto the Chromium Single Cell Controller using the Chromium Single Cell 3′ Library & Gel Bead Kit v2 (both from 10X Genomics) chemistry following the manufacturer’s instructions. Sample processing and library preparation was performed according to manufacturer instructions using AMPure beads (Beckman Coulter). Sequencing was carried out on a local Illumina Nextseq 500 using the High-Out 75 cycle kit with a 26-8-0-57 read setup.

### Code reproducibility

The code for reproducing the results in this manuscript has been deposited at https://github.com/chenlingantelope/MSscRNAseq2019.git.

### Preprocessing of sequencing data

Processing of sequencing data was performed with the *cellranger* pipeline v2.0.2 (10X Genomics). Raw bcl files were demultiplexed using *cellranger mkfastq*. Subsequent read alignments and transcript counting was done individually for each sample using *cellranger* count with standard parameters. *Cellranger aggr* was employed, to ensure that all samples had the same number of confidently mapped reads per cell. The *cellranger* computations were carried out at the High Performance Computing Facility of the Westfälische Wilhelms-University (WWU) Münster.

### Single-cell sample filtering

Initial exploratory data analysis identified one MS sample and one IIH sample whose clustering did not overlap with any of the other samples. This suggested strong batch effects. Both samples were excluded from further analysis, leaving four control- and four MS-derived samples from CSF, and five control and five MS-derived samples from PBMC.

Nine barcode-level quality control (QC) metrics were computed for the unfiltered 10x Cell Ranger output: (1) number of unique molecular identifiers (UMIs), (2) number of reads, (3) mean reads per UMI, (4) standard deviation of reads per UMI, (5) percent of reads confidently mapped to the gene, (6) percent of reads mapped to the genome but not a gene, (7) percent of reads unmapped, (8) percent of UMIs corrected by the Cell Ranger pipeline, and (9) the number of cell barcodes corrected by the Cell Ranger pipeline. These metrics were used for filtering and normalization. We applied the gene and sample filtering using a scheme. involving four steps^71^:

1. Define common genes based on UMI counts: genes with *n*_u_ or more UMIs in at least 25% of barcodes, where *n*_u_ is the upper-quartile of the non-zero elements of the UMI matrix.
2. Filter samples based on QC metrics. Remove samples with low numbers of reads, low proportions of mapped reads, or low numbers of detected common genes. The threshold for each measure is defined data adaptively: a sample may fail any criterion if the associated metric under performs by *z*_cut_ standard deviations from the mean metric value or by *z*_cut_ median absolute deviations from the median metric value. Here, we have used *z*_cut_ = 2. This function is implemented in scone::metric_sample_filter (see below).
3. Remove barcodes from donors with fewer than 100 barcodes following sample filtering. These donors have contributed too few high-quality samples to reliably estimate donor-specific effects. Only seven cells were removed in this step.
4. Filter genes based on UMI counts: genes with *n*_u_ or more UMIs in at least *n*_s_ barcodes, where *n*_u_ is the upper-quartile of the non-zero elements of the sample-filtered UMI matrix. We have set *n*_s_ = 5 to accommodate markers of rare populations. This sub-step ensures that included genes are detected in a sufficient number of samples after sample filtering. For the CD4^+^-only analysis this step was applied again after the data matrix was subset to include only CD4^+^ clusters.

### Single-cell harmonization

We utilized a Bayesian variational inference model scVI^28^ to infer a shared latent space of dimension ten for all single cells from different tissue, condition, and batches. Visualizations were generated using Uniform Manifold Approximation and Projection (UMAP) to further reduce the latent space to two dimensions. scVI is a deep generative model that learns a probabilistic representation of the transcriptional states of single cells conditional on the sequencing batches, thus no explicit library size and batch correction is needed.

### Level 1 clustering analysis

After sample filtering, we performed louvain clustering on the scVI latent space as implemented in https://github.com/taynaud/python-louvain. We first constructed a *k*-nearest-neighbor graph from the scVI latent space, and then used the louvain.find_partition function with the ModularityVertexPartition method to recover a total of 25 clusters. Three of these clusters correspond to CD4 T cells and were tentatively combined into a single cluster for further analysis resulting in 22 first level clusters (Supplementary Fig. 2a, b). From this, we removed one RBC cluster (2333 cells; *HBA1*, *HBA2*, and *HBB*), three clusters with high doublet probability (see below), and one blood-derived cluster with low quality (mitochondrial genes, no canonical marker genes; 361 cells) for further analysis (Supplementary Fig. 2a, b).

### Doublet detection

We computed a doublet score for each single cell using the function scrub_doublets in the Scrublet package with all default parameters. We then removed all clusters with >20% of cells labeled as doublets (1186, 290, and 105 cells), including one cluster of lower quality cells, one cluster expressing Monocyte marker genes, and one cluster expressing B cell marker genes.

### Level 2 clustering analysis

For cells that were classified as a single cluster but two distinct clusters were visible on UMAP visualization (monocytes, B cells and mDCs; Supplementary Fig. 2b, c), we performed further clustering on the scVI latent space using Spectral Clustering from the scikit-learn package SpectralClustering with number of cluster set to 2 and affinity matrix computed using *k*-nearest-neighbor with *k* = 15 (Supplementary Fig. 2c). The clusters we visually identified on UMAP were confirmed to be the same as the results of Spectral Clustering. With further validation using signature genes, we included the second level clusters into the main analysis. Monocyte cluster separated into Mono1 and Mono2, B cell cluster separated into clusters B1 and B2, and mDC separated into mDC1 and mDC2.

### T cell clustering analysis

For all CD4 T cells (excluding regulatory T cells), we performed Louvain clustering on the scVI latent space, excluding all other cells. With the same parameters as the level 1 clustering analysis. We partitioned the CD4 T cells into a total of 12 clusters.

### Systematic comparison with published microglia and CSF datasets

We obtained the key marker genes of myeloid lineage cell clusters from recent publications^26,54,72,73^ and plotted their expression onto our combined dataset (Supplementary Fig. 14). We extracted the combined oligodendrocyte markers from a study performing single nuclei RNA-seq of frozen brain parenchyma^72^ and selected genes that are also highly variable in our dataset (genes *APOE*, *CD74*, *HLA-DRA*, *PTPRC*, and *C3*). We extracted markers of five myeloid clusters from a CSF-based study^54^ (genes *C1QB*, *C1QC*, *APOE*, *C1QA*, *LYVE1*, *SEPP1*, *FCGBP*, *APOC1*, *C3*, *A2M*, *MSR1*, *EPB41L2*, *MARCO*, *RNASE1*, and *F13A1*). We also obtained microglia markers (*TMEM119*, *CCL4*, P2RY13, *EGR2*, *CX3CR1*, *CCL2*, *SLC2A5*, *EGR3*, and *CD83*) and markers of MS-associated microglia markers (CTSD, CD74, SPP1, APOC1, HLA-DRA, PADI2, GPNMB, HLA-DRB1, ANXA2, HLA-DPB1, CPM, LGALS1, LYZ, LIPA, APOE, and MAFB)^26^. We extracted marker genes from a rodent study (NLRC5, IL12RB1, PSMB9, TAP1, TAP2, IFIH1, IRF7, and ZBP1)^73^. We then plotted the combined expression level of the respective gene signatures into our combined blood and CSF dataset.

### VISION analysis

We passed raw and normalized UMI data to the VISION pipeline (https://github.com/YosefLab/VISION)^41^. Mean expression per gene symbol was calculated prior to the analysis in order to make the features relatable to general gene signatures. The goal of FastProject analysis—on which VISION is based—is to uncover biologically meaningful gene signatures that vary coherently across single-cell neighborhoods^41^. These signatures can help assign meaning to the dominant expression differences between clusters. In addition to raw data, we passed QC, donor, status, and Seurat cluster covariates for exploratory analysis and visualization. VISION quantifies the extent to which cell signature values cluster across the cell manifold by using consistency testing. VISION scores the extent to which neighbouring cells (similar expression profiled) are predictive of a cell’s signature value using autocorrelation (Giri’s C) statistics, comparing against random permutations in order to assign statistical significance with respect to a uniform null model. We also included the Seurat *t*-SNE as a precomputed projection. Our signature set includes:

1. Human cell cycle genes described before^7^, representing sets of genes marking G1/S, S, G2/M, M, and M/G1 phases.
2. The MSigDB C7 immunological signature collection.
3. T_H_ signatures compiled previously^45^.
4. NetPath database signatures.
5. Curated T cell signatures^42^.
6. Curated TFH signatures^46^.
7. Curated Temra signatures^43^

Housekeeping genes were referenced from ref. ^74^.

### Differential composition analysis

For both the initial and the CD4^+^-only clustering, we used *t*-test and beta-binomial generalized linear model in package aod::betabin to test the difference in cluster abundances (cell counts) between MS donors and control donors. We used both methods because when cell types are rare, the estimated proportions of a cell type in each donor might be over-dispersed. The two methods show consistent results and thus we show the differential composition analysis from the beta-binomial distribution comparison. For the beta-binomial regression model unless indicated in the figure legends, we set the count of the cell type of interest and the total count of cells of each donor to be the response variable and the state of the donor (MS or control) or the tissue of origin (CSF or blood) to be the independent variable. We tested for Pearson’s correlation between the frequency of each B cell cluster and cluster 0 in CD4 T cells. We adjusted the *p* value threshold to 0.05/15 = 0.0033, since we tested for significant correlation using three B cell subsets in five different sample partitions (all samples, CSF only, blood only, MS only, and control only). The abundance of cluster 0 in CD4 T cells is not signifiantly correlated to B cell subset abundances in any of these comparisons.

### Differential expression analysis

We used three different tests for the discovery of differentially expressed genes between two groups of cells. First, we computed Bayes factor using the imputed counts from scVI. Bayes factor is a generalization of the *p* value and is computed as *λ* where *x*_*a*_ is the gene expression of the gene of interest in group *a* and *x*_*a*_ in group *b*. We use the generative model of scVI to obtain the batch-corrected mean of the negative binomial distribution of transcript counts. Second, we used the library-size corrected UMI counts for Mann–Whitney *U* test. At last, we followed the methods of the best performing method in a single-cell specific DE method assessment paper^75^ and we used EdgeR^27^ with cellular detection rate and batch id as covariates.

### GSEA

After deriving lists of differentially expressed (DE) genes (Supplementary Datasets 2, 4, 5, 7, and 10), we applied GSEA tests^76^ to all cluster specific DE gene lists DE between CSF and blood (Supplementary Dataset 3 and  8). We used the enrichr function in gseapy v0.9.12 to find overlap between the DE genes and function genesets. We used signed significance scores based on the Adjusted *p* value provided by the enrichr function. Sets considered in this analysis include all MSigDB C7 signature sets and all curated T cell signature sets described previously^42^ with ten or more genes quantified in the present study; “UP” and “DN” signature subsets were tested separately.

### CSEA

For the CD4^+^ T cells analysis, we developed a novel adaptation of the GSEA method, applying the technique to cell sets: CSEA (illustrated in Supplementary Fig. 9a, representative workflow and code in Supplementary Dataset 11). CSEA is a hypothesis testing method for simultaneously uncovering enrichments and identifying subsets of cell sets of importance. In this procedure, a collection of cells is first ordered by a transcriptional phenotype of interest (e.g., sum expression of genes in a pathway). The resulting statistical test is sensitive to cases in which only a subset of cells from one group (e.g., MS) exhibit unusually high values of the transcriptional phenotype. The input to this method is a list of *N* cells, rank-ordered by some input signal. Our analysis uses VISION signature scores, reflecting known axes of biological variation. VISION signature scores—based on FastProject signature scores^41^—are computed by first centering and scaling each normalized log expression cell profile. Following scaling, the sum of gene expression values in the negative signature subset are subtracted from the sum of gene expression values in the positive signature subset. Signatures are normalized to the total number of genes in the set. For example, a signature set that describes a dichotomy between naive and memory T cells may be used to score individual cells, indicating that some cells have higher expression of genes characterizing the naive state and lower expression of genes characterizing the memory state. Using the notation^76^ we will use *r*_j_ to denote the cell j’s signature score; indices have been sorted so that *r*_j_ > *r*_j+1_. The test involves considering all cells up to a specific position, i. A “hit” score is defined as the signature score optionally exponentiated by parameter *p* (|*r*_j_|^*p*^) for members of cell set *S*, divided by the sum over all set members in the list. A “miss” score is similarly calculated for nonmembers of *S*, but without weighing by signature score magnitudes.

The CSEA ES is defined as the maximum of the difference between the running cumulative sum of hit scores and miss score with respect to index i. When *p* = 0, the ES reduces to a one-sided KS test statistic for differential signature analysis between cell sets. When *p* = 1, the cells in *S* are weighted by their signature score, normalized by the sum of the score over all the cells in *S*. We apply the same permutation scheme as described for GSEA above. For *p* > 0, CSEA cannot be seen as a simple differential signature test: CSEA tests for enrichment of a cell set at the high tail of the signature score distribution, but additionally weighs the set elements according to their signature value. This reduces the effects of low-magnitude cells in *S*, whereas all cells not in *S* are treated the same no matter the magnitude of their signature score. CSEA tests if high magnitude (positive or negative) cells are enriched at a specific tail, applying permutation tests to account for the additional variability induced by the magnitude weights. The set of indices up to where the objective score reaches its maximum also holds significance—in GSEA^76^ referred to as the “leading edge” of the enrichment test. The intersection of the set *S* and the leading edge is the leading edge subset, representing an important core subset of cells driving an enrichment. For each VISION signature, we treated the computed signature scores as cell signature scores *r*_j_. The sets under consideration were the mutually exclusive sets of MS and control cells. The goal of this approach is to identify core sets of cells that drive each biological condition’s enrichment for high signature values (Supplementary Fig. 9).

To screen a set of gene signatures, we computed the Vision signature score for 64 gene signatures related to CD4 T cell states, cell cycle, IL expression, and T cell subsets (Supplementary Dataset 9). To determine the *p* value of the CSEA ES for a geneset, we shuffle the disease state labels for cells 100 times and compute the probability that the maximum ES computed with the true labels is greater than the ES computed with the shuffled labels. We also generated a random geneset that is matched to the original signature set in both number of genes and the average expression of each gene. This is done by finding the top 20 gene that has the closest mean expression to each gene in the original signature set, and then randomly sampling one of them. We then corrected for multiple testing using the Benjamini–Hochberg procedure to generate the corrected *p* values. We filtered the result based on three criteria: the corrected *p* value of the true signature set is smaller than 0.05, the corrected *p* value of the control signature set is >0.05, and that the leading edge is <1000. This results in two significant signatures, TFH and Th1. We then validated this result by computing the ES of 1000 matched control genesets for each signature set. We then report the *p* value as the probability that the true signature set’s maximum ES being greater than the maximum ES in the matched random genesets. We also computed the ES for enrichment in control and found that the ES is significantly larger than the control set but the leading edge is much larger than for enrichment in CSF.

We also tested the performance of our model on varying match levels of the randomized geneset to the original signature set (Supplementary Fig. 13). The match levels do not affect the results significantly, showing that our conclusion is not driven by the gene-matching procedure itself. However, when randomized genesets are selected completely randomly, the ES become extremely variable, showing that some degree of matching is required. We validated our method through two simulation schemes (Supplementary Fig. 15). First, we directly simulate a continuous distribution of cell states by drawing from a mixture of Gaussian distributions. The control group cells is drawn from a *N(5, 1)* Gaussian distribution, and the disease group is drawn mostly from the same distribution, with a small proportion *p* drawn from a *N(mean2, 1)* Gaussian distribution. When we set mean1 to be equal to mean2, the *p* value distribution is close to a uniform distribution. We expect the detection of the outlier population to be more difficult when the distributions are close to each other (mean2 close to 5), and when the outlier population is small. We run CSEA on this simulated data, and show that if the two distributions mean are 2 standard deviations apart, CSEA can correctly detect an outlier population when the outlier population is >5% of the total population. If we hold the outlier population frequency at 10%, we can detect the outlier up to when the two distribution means are 1 standard deviations apart from each other.

The second simulation scheme is through a single-cell specific simulation software called SymSim. We also simulate a continuous variable corresponding to cell state using the evf_type = “continuous” option in SymSim. Instead of simulating directly the cell state, we first simulate a gene-cell matrix and compute the score of each cell on the continuous trajectory based on their expression of differentially expressed genes. Cells are assigned to the case or control group by matching each cell with a cell in the previous simple simulation by their rank. The results of this more realistic simulation is similar to the previous one: when the two cell populations are not different from each other, CSEA *p* values are uniformly distributed. CSEA detects the outlier population in most replicates if the two distributions mean are 2 standard deviations apart, and the outlier population is >6% of the total population. If we hold the outlier population frequency at 10%, we can detect the outlier up to when the two distribution means are 1 standard deviations apart from each other. A more comprehensive description and validation of this method is being prepared for publication by Arpita Singhal and Chenling Antelope.

### Bulk RNA-seq of sorted TFH cells

TFH cells were sorted from the CSF of nine MS donors and nine IIH donors using a BD FACS Aria III cell sorter using an 85 µm nozzle and the drop delay was determined using BD Accudrop beads. Sorting was performed using sort precision mode “purity” for live CD3^+^CD4^+^CXCR5^+^ cells. Antibodies against CD3 (UCHT1), CD4 (OKT4), CXCR5 (J252D4), PD-1 (EH12.2H7), and ICOS (C398.4 A) were from Biolegend. Cells were sorted directly into 1.5 ml reaction tubes containing 100 µl RNA Lysis Buffer (Zymo Research). After sorting, tubes were vortexed, briefly centrifuged and frozen at −80 °C until RNA isolation. Data were analyzed using FlowJo software v10.4.1 (Tree Star, Inc.). Samples for bulk RNA-seq were prepared using a modified version of the SmartSeq2 protocol^77^. Briefly, unquantified purified RNA was used as input. Reaction volumes were scaled up and the number of PCR cycles during cDNA amplification adjusted accounting for the higher number of input cells compared to the original protocol^77^. Library Preparation was done by the Next Ultra II FS DNA Library Prep Kit (New England Biolabs) using 1–3 ng of cDNA as input. Sequencing was carried out on a NextSeq500 using the High-Out 75 cycle kit (Illumina).

### Bulk expression quantification

RNA-seq reads were aligned to the RefSeq hg38 transcriptome (GRCh38.2) using Bowtie2. The resulting transcriptome alignments were processed using the RNA-Seq by Expectation Maximization (RSEM) toolkit to estimate expected counts over RefSeq transcripts. Several genes were quantified multiple times due to alternative isoforms unrelated by RefSeq annotation. Before expression data normalization, the gene entry with maximum counts was selected to represent the gene in further analysis.

### Bulk RNA-seq data analysis

Sample and gene filtering were similar to the scRNA-seq filtering method above, enforcing (>107k reads, >10% read alignment (forced), >93.3% common genes detected; corresponding to *z*_cut_ = 20). Out of 18 initial samples (9 control vs. 9 MS), 5 total samples (3 control vs. 2 MS) were removed after QC. Setting *n*_s_ = 1, we analysed 11,383 genes below.

For each sample, we computed transcriptome alignment and quality metrics using FastQC (Babraham Bioinformatics), Picard tools (Broad Institute), and custom scripts. Computed metrics included: (1) number of reads; (2) number of aligned reads; (3) percentage of aligned reads; (4) number of duplicate reads; (5) primer sequence contamination; (6) average insert size; (7) variance of insert size; (8) sequence complexity; (9) percentage of unique reads; (10) ribosomal read fraction; (11) coding read fraction; (12) UTR read fraction; (13) intronic read fraction; (14) intergenic read fraction; (15) mRNA read fraction; (16) median coefficient of variation of coverage; (17) mean 5′ coverage bias; (18) mean 3′ coverage bias; and (19) mean 5′–3′ coverage bias.

Data were normalized using SCONE. 569 positive controls were derived from MSigDB C7 entries annotated to include TFH cell types, including the most frequently included gene symbols in those entries. Negative controls for RUVg and evaluation were derived from the housekeeping gene list. Control lists were sampled down to 186 genes per list so as to match mean expression of genes in each list. The study group included two batches with 4/3 and 3/3 MS/IIH samples, respectively. Biological condition was used only for evaluation. SCONE recommended TMM (trimmed mean of M values) scaling and adjustment for two factors of RUVg and batch condition.

We performed PCA on the scaled log-transformed normalized data for visualization. DE between MMS and IIH donors was performed with limma-voom, using RUVg factors and batch in the model to adjust for unwanted variation. Per-gene DE significance scores were computed from log-transformed *p* values. No single gene reached significance after correction for multiple hypothesis testing. The 42 most frequent core members of the significant enrichments (Bonferroni adjusted *P* < 0.01)—genes driving 7 or more of these enrichments—were selected and their normalized log values were correlated against each-other and represented in a sorted heatmap using *pheatmap* defaults.

### Mice and EAE induction

CD4^*Cre*^ (ref. ^78^), 2D2.^tg^ (ref. ^53^), and B6.129 S(FVB)-*Bcl6*^*tm1.1Dent*^/J (named Bcl6^*flox*^ or Bcl6^*fl/fl*^) mice^51^ were purchased from the Jackson laboratories. The CD4^*Cre*^Bcl6^*flox*^ strain was maintained by breeding the Bcl6^*flox*^ allele to homozygosity (i.e., Bcl6^*fl/fl*^) and breeding the Cre alleles in heterozygous to wild-type matings. The mice used in the experiments were littermates and were on a pure C57BL/6 J genetic background. Genotyping was done by routine PCR from ear punch DNA. The animal research protocols were approved and supervised by the responsible state authorities (Landesamt für Natur, Umwelt und Verbraucherschutz (LANUV), English: ‘State Agency for Nature, Environment and Consumer Protection’ of the German state North Rhine-Westphalia (NRW)) under reference number 84-02.04.2015.A319 and were performed in accordance with local regulations from the “Tierschutzbüro der Medizinischen Fakultät der Westfälischen Wilhelms-Universitat Münster” (English: Animal Protection Office of the Medical Faculty of the Westfälische Wilhelms-University Münster). Mice of both sexes (8–14 weeks old) were immunized s.c. in the flanks with an emulsion containing the MOG peptide MOG_35–55_ (150 μg/mouse; GL Biochem (Shanghai) Ltd) and *Mycobacterium tuberculosis* H37Ra extract (5 mg/ml, BD) in CFA (complete Freundʼs adjuvant; 200 μl/mouse). Pertussis toxin (250 ng/mouse, Sigma) was administered intraperitoneally on days 0 and 2.

Adoptive transfer EAE induction was performed by flow sorting naive CD44^low^CD62L^high^ CD4^+^ T cells from 2D2^tg^ donor mice and culturing at 2 × 10^6^ per ml in the presence of irradiated antigen presenting cells, soluble anti-CD3 antibody (2.5 μg/ml), IL-6 (20 ng/ml), TGF-β1 (10 ng/ml), and anti-IFNγ antibody (10 μg/ml) for 2 days (all cytokines were purchased from R&D). Cells were subsequently split when necessary using IL-23 (10 ng/ml) containing media for three additional days and then plated at 2 × 10^6^ per ml onto plates coated with anti-CD3 and soluble anti-CD28 (both at 2 μg/ml) in the absence of cytokines for 2 days. Cytokine production was assessed on day 5 after initial plating. Two days later, 5 × 10^6^ total cells were intravenously injected into C57BL/6 recipients.

Mice were monitored daily and assigned grades for clinical signs of EAE using the following scoring system: 0, healthy; 1, paralyzed tail tip; 2, paralyzed tail; 3, waddling; 4, hind legs drag on the ground; 5, butt on the ground; 6, one paralyzed hind leg; 7, both paralyzed hind legs; 8, one paralyzed front leg (criterium to stop EAE); 9, both paralyzed front legs; and 10, moribund or death. Detailed refinement procedures were performed according to the impairments of the mice. Mice with a score of >7 were euthanized. Additionally, adoptive transfer EAE recipient mice were scored with an ataxia scoring system consisting of the following criteria: ledge test, hindlimb clasping, gait, and kyphosis^79^. Every criteria was rated on a scale from 0 (healthy) to 3 and all points were added up to a maximum ataxia score of 12. GraphPad Prism 5 was used for statistical analysis of all mouse-related data.

### Isolation of CNS-infiltrating mononuclear cells and lymphoid tissue characterization

Mice were intracardially perfused with cold phosphate-buffered saline (PBS). The forebrain and cerebellum were dissected and spinal cords flushed out from the spinal canal with hydrostatic pressure. CNS tissue was cut into pieces and digested with collagenase D (2.5 mg/ml, Roche Diagnostics) and DNase I (0.05 mg/ml, Sigma) at 37 °C for 20 min. Mononuclear cells were isolated by passing the tissue through a 70 μm cell strainer, followed by a 70%/37% percoll gradient centrifugation. The interphase was removed, washed, and cells were stained at room temperature for 30 min with anti-mouse antibodies (Biolegend, clones indicated): CD45 (30-11 F), CD3 (17A2), CD4 (RM4-5 or GK1.5), B220 (RA3-6B2), CD19 (6D5), and live/dead staining “Zombie NIR” (Biolegend; 1:500) in PBS. Lymph node and spleen cells were additionally stained using CD8 (53-6.7), CD11b (M1/70), CD11c (N418), Gr1 (RB6-8C5), and NK1.1 (PK136) antibodies. For in vitro Th cell differentiation, naive CD44^low^CD62L^high^ CD4^+^ T cells were FACS sorted and cultured at 10^6^ per ml for 4 days with coated anti-CD3 antibody (2 µg/ml), soluble anti-CD28 antibody (2 μg/ml) and (A) IL-6 (20 ng/ml), TGF-β1 (10 ng/ml), and anti-IFNγ antibody (10 μg/ml) for Th17 differentiation, (B) IL-12 (20 ng/ml) for Th1 differentiation, or (C) TGFβ1 (5 ng/ml) for Treg differentiation. To determine cytokine production, cells were resuspended in culture medium containing 20 ng/ml PMA, 500 ng/ml ionomycin, GolgiStop, and GolgiPlug (BD, each 1:1000 diluted). After 4 h of incubation at 37 °C, cells were stained extracellularly, fixed, and permeabilized using the Foxp3/Transcription Factor Staining kit (eBioscience) according to the manufacturer’s protocol. Afterward, IL17A (eBioscience, eBio17B7), IFNγ (Biolegend, XMG1.2), and FoxP3 (eBioscience, FJK-16s) were stained intranuclearly/intracellularly. Cells were washed and analysed using a Gallios flow cytometer (Beckman Coulter) and analysed using FlowJo V10.

### Histology

For histology, mice were intracardially perfused with 20 ml cold PBS and fixed by perfusion with 10 ml of 4 % paraformaldehyde (PFA). Spinal cords were removed and kept in PFA for 48 h at 4 °C. The fixed spinal cords were cut into 3 mm thick transverse segments and embedded in paraffin. To evaluate demyelination, spinal cord sections were stained with Luxol Fast Blue and subsequently incubated with Periodic acid-Schiff. Immunohistochemistry was performed using the biotin-streptavidin peroxidase technique (K5001, Dako) in an immunostainer (AutostainerLink 48, Dako). Sections were pretreated in a steamer (treatment solutions pH 6.0 or pH 9.0 (Dako)) before incubation with the primary antibodies against CD3 (clone CD3-12, BioRad, 1:100) or Mac3 (clone M3/84, BD, 1:100) or B220 (clone RA3-6B2, BD, 1:200). DAB (3,3ʼ-Diaminobenzidin) was used as a chromogen. For B220/Ki67 double-immunofluorescence staining, B220 (clone RA3-6B2, BD, 1:100) and Ki67 (clone SP6, Thermo Scientific, 1:100) were used as primary antibodies; AF488- and AF594-labeled secondary antibodies (both 1:100) were used for visualization. Stained sections were analysed with a keyence microscope and pictures were taken with an Axioplot camera. ImageJ v1.48 was used to manually count infiltrated cells and measure areas.

### Reporting summary

Further information on research design is available in the Nature Research Reporting Summary linked to this article.

## Supplementary information

Supplementary Information

Dataset 1

Dataset 2

Dataset 3

Dataset 4

Dataset 5

Dataset 6

Dataset 7

Dataset 8

Dataset 9

Dataset 10

Dataset 11

Reporting Summary

## Data Availability

Raw bulk RNA-seq data from the present study have been deposited in GEO repository with the accession code GSE141797 and raw single-cell RNA-seq data from the present study have been deposited in GEO repository with the accession code GSE138266. All processed, unmodified scRNA-seq data (differential expression data, GSEA, and CSEA data) are included as Supplementary Dataset Tables. Technical scRNA-seq information and data tables with details of the included patients are included as Supplementary Tables. The source data underlying Figs. 3f, g, 4b–f and Supplementary Figs. 1a–e, 4c, d, 10b–f are provided in the Source Data file.

## Source data

source data

## Code availability

The code for reproducing the single-cell sequencing results in this manuscript has been deposited at https://github.com/chenlingantelope/MSscRNAseq2019.git
