# Supplementary material for: Integrated single cell analysis of blood and cerebrospinal fluid leukocytes in multiple sclerosis
Source: Nat Commun. 2020 Jan 14;11:247. doi: 10.1038/s41467-019-14118-w (PMC6959356; doi:10.1038/s41467-019-14118-w)
Supplement: Supplementary file 1 — Supplementary Information [file 41467_2019_14118_MOESM1_ESM.pdf]

Supplementary Information

Integrated single cell analysis of blood and cerebrospinal fluid leukocytes in multiple sclerosis

Schafflick *et al.*

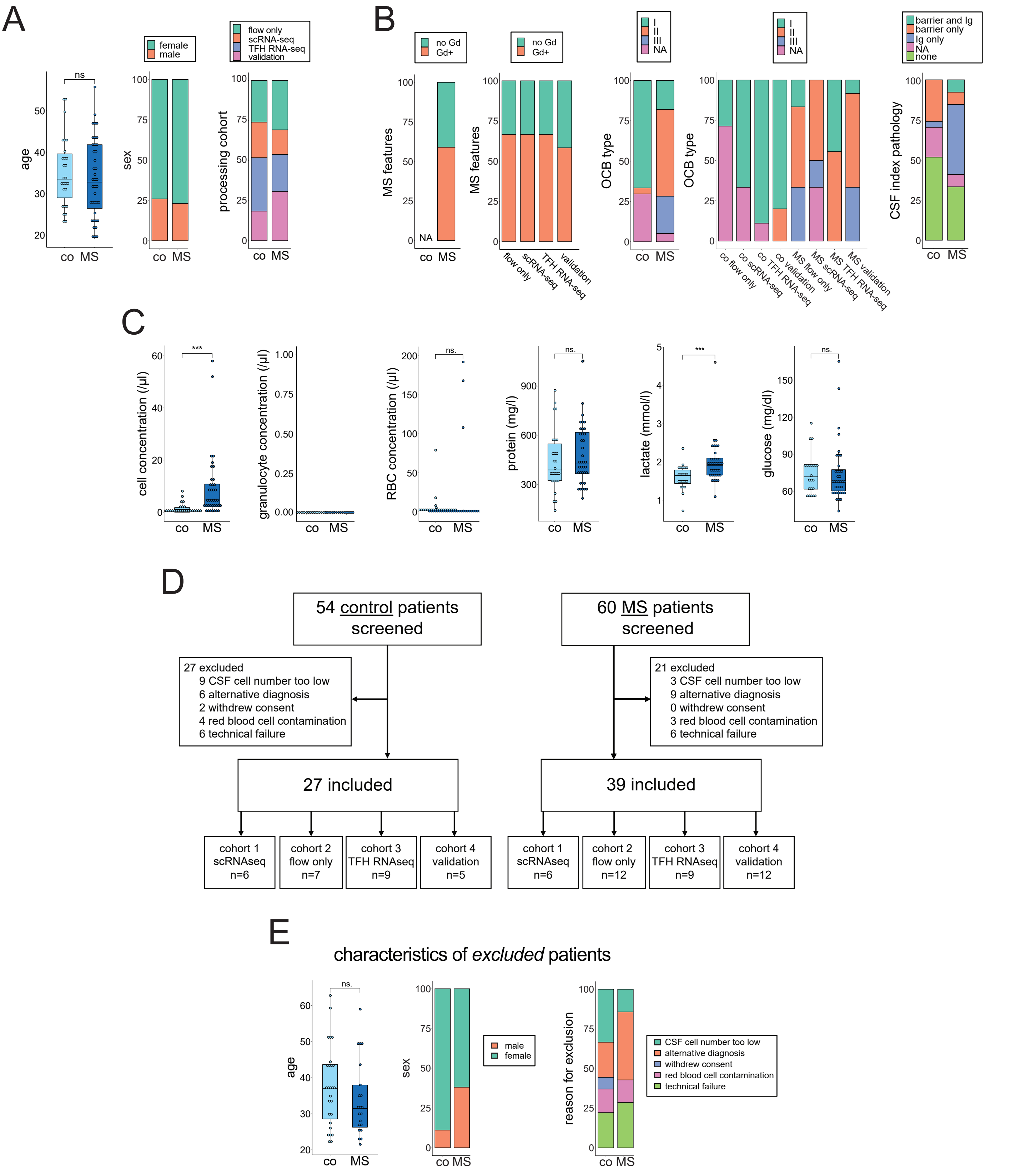

Supplementary Figure 1: Patient characteristics.
(A) Age and gender of all control (co, n = 27) and multiple sclerosis (MS, n = 39) patients included in the study are depicted. (B) Disease characteristics of MS patients. MS patients were classified to either have (Gd+, Gadolinium) or not have (no Gd) contrast enhancing lesions in brain or spinal cord detected by magnetic resonance imaging. Oligoclonal bands (OCB) in CSF were classified as being either undetectable (type 1), restricted to CSF (type 2), detected in serum and additionally in CSF (type 3), or not determined (?). CSF/serum indices for albumin and immunoglobulin G (IgG) were calculated. The CSF barrier function (CSF index pathology) was evaluated as being either unaffected (none), showing intrathecal IgG synthesis (Ig only), showing barrier dysfunction (barrier only), or showing both intrathecal IgG synthesis and barrier dysfunction (barrier & Ig). (C) Standard CSF parameters of all included study patients. *RBC* red blood cells. (D) The study recruitment scheme is depicted. 50% of control and 35% of MS samples were excluded after screening for the reasons indicated. Samples from all patients were divided into four cohorts by time of recruitment and all samples were analysed by flow cytometry (Suppl. Tab. 1 and Methods). (E) Clinical characteristics (age, sex) of patients excluded after screening and reasons for exclusion are shown. *NA* not applicable. Mann-Whitney U test, *P < 0.05, **P < 0.01, ***P < 0.005. The lower and upper edges of the box plots represent the lower and the upper quartile, respectively, the horizontal line inside the box indicates the median, and the whiskers extend to the most extreme values within the 1.5 interquartile range of the lower/upper quartile. Source data listing all clinical parameters are provided in the Source Data file and Supplementary table 2.

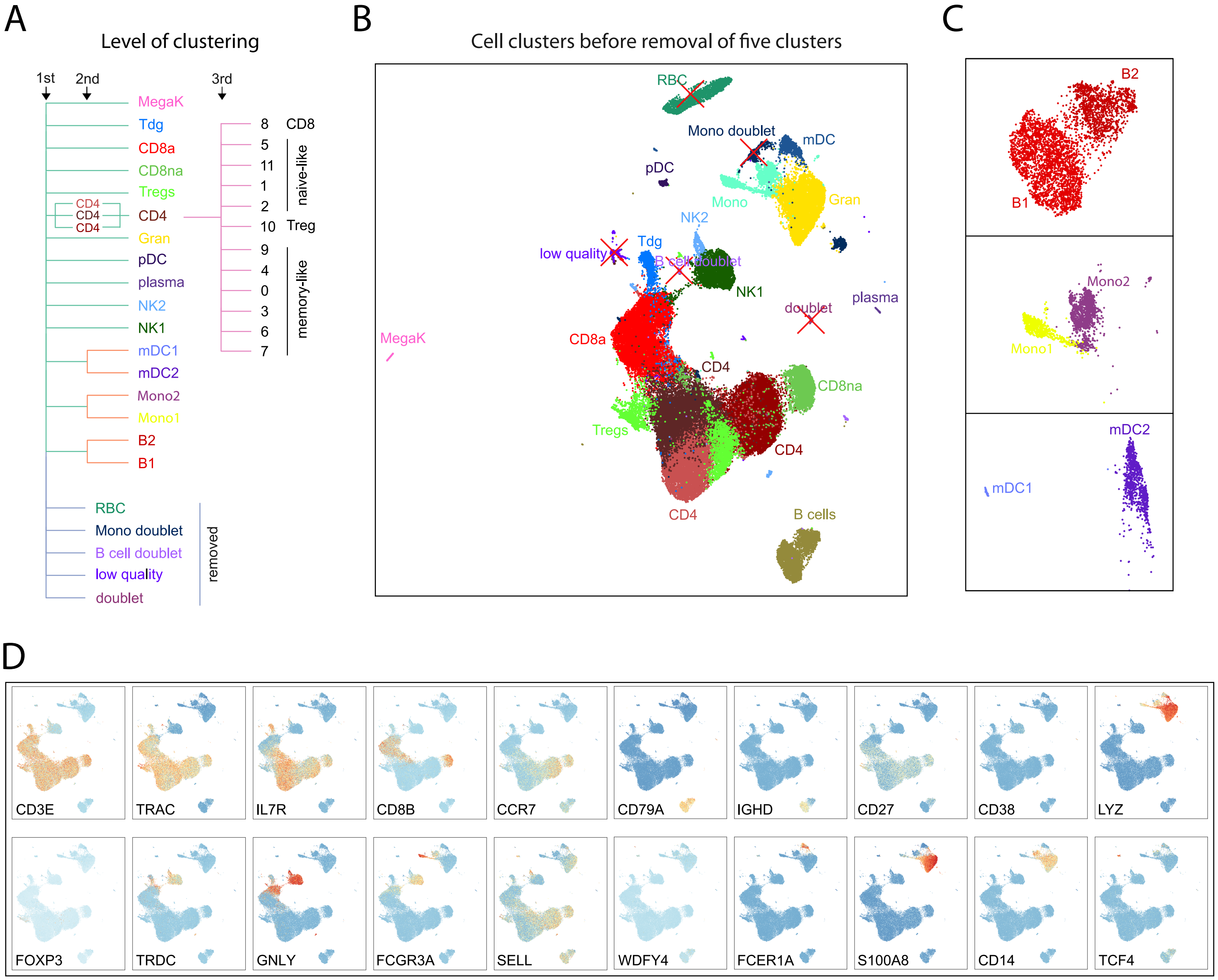

Supplementary Figure 2: Details on the clustering approach and cell cluster identification based on scRNA-seq data.
(A) First (1st) level clustering (see panel B) returned 22 cell cluster after tentatively merging CD4^{+} T cell (CD4) subclusters. Doublet, low quality, and red blood cells (RBC) were removed. Second (2nd) level clustering was performed on B cell (Bc), monocyte (mono), and mDC clusters (see panel C). Third (3rd) level clustering refers to sub-clustering of CD4^{+} T cells (see Fig. 2). Color codes correspond to Fig. 1B. (B) UMAP plot depicting first level clustering of all combined blood and CSF single cell transcriptomes. (C) UMAP plots showing 2nd level sub-clustering of B cells (top), monocytes (middle), mDC (bottom). (D) Feature plots of selected marker genes as in Fig. 1B. Cluster key as in Fig. 1.

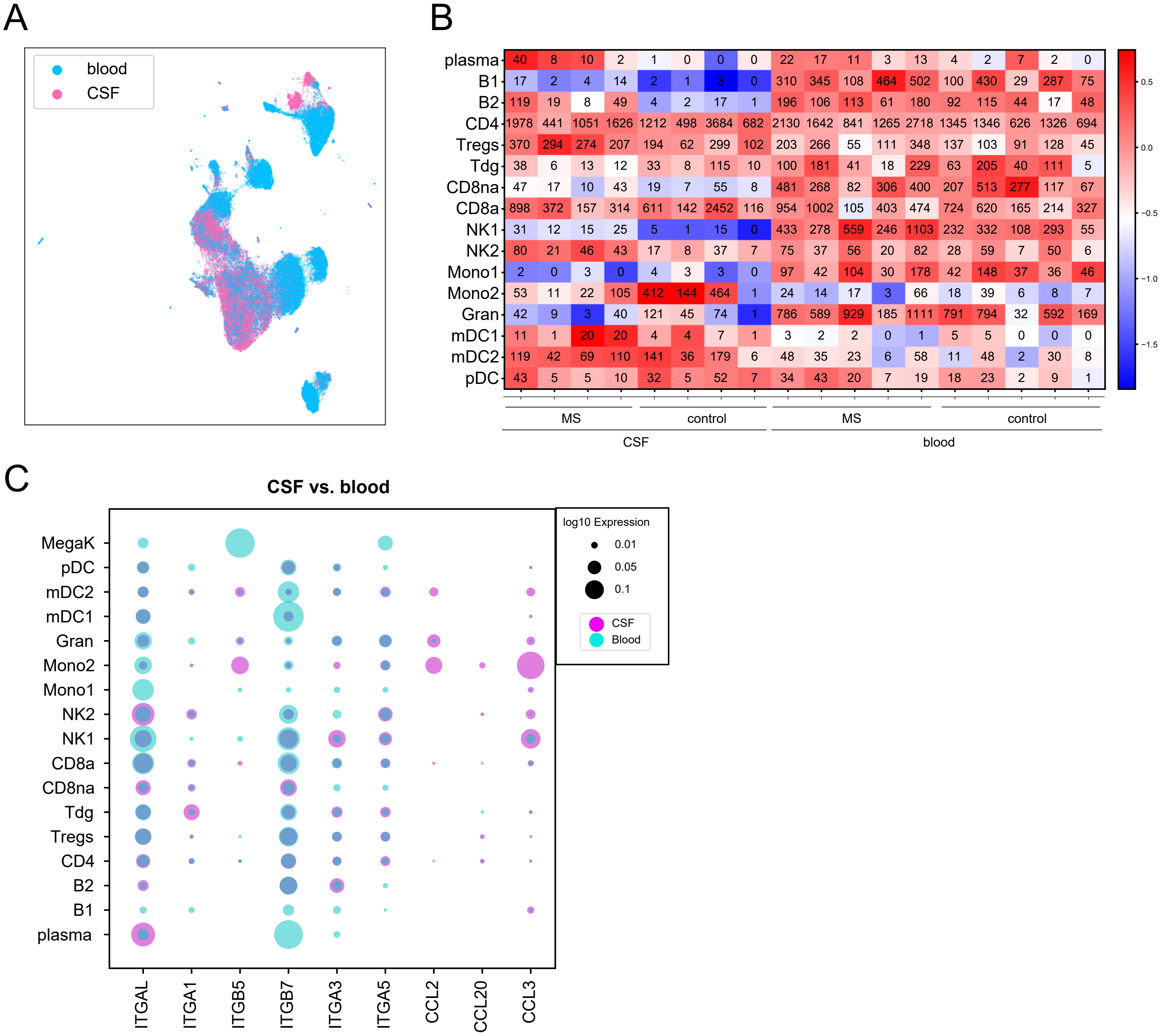

Supplementary Figure 3: Inter-individual donor heterogeneity of cell cluster abundance.

(A) UMAP plot depicting all cells shown in Fig. 1A color-coded by tissue of origin after 2nd level clustering with blue indicating blood cells and purple indicating CSF cells. (B) Heatmap depicting cell numbers in each cell cluster in each donor sorted by tissue of origin and by disease-status. Numbers in the heatmap represent cell numbers in each cluster after 2nd level clustering. Color code indicates the relative abundance of cell types per donor, compared to the row average. (C) Dotplot depicting selected genes differentially expressed in at least one cluster of CSF cells compared to blood. Purple dot size encodes the average expression in CSF and turquoise dot size encodes the average expression in blood. Dots are partially transparent thus overlap is dark blue. Purple edge around dark blue circle indicates higher expression in CSF, while turquoise edge around dark blue circle indicates lower expression in CSF compared to blood. Source data for (C) listing the differential expression values for CSF vs. blood are provided in Supplementary Dataset 2.

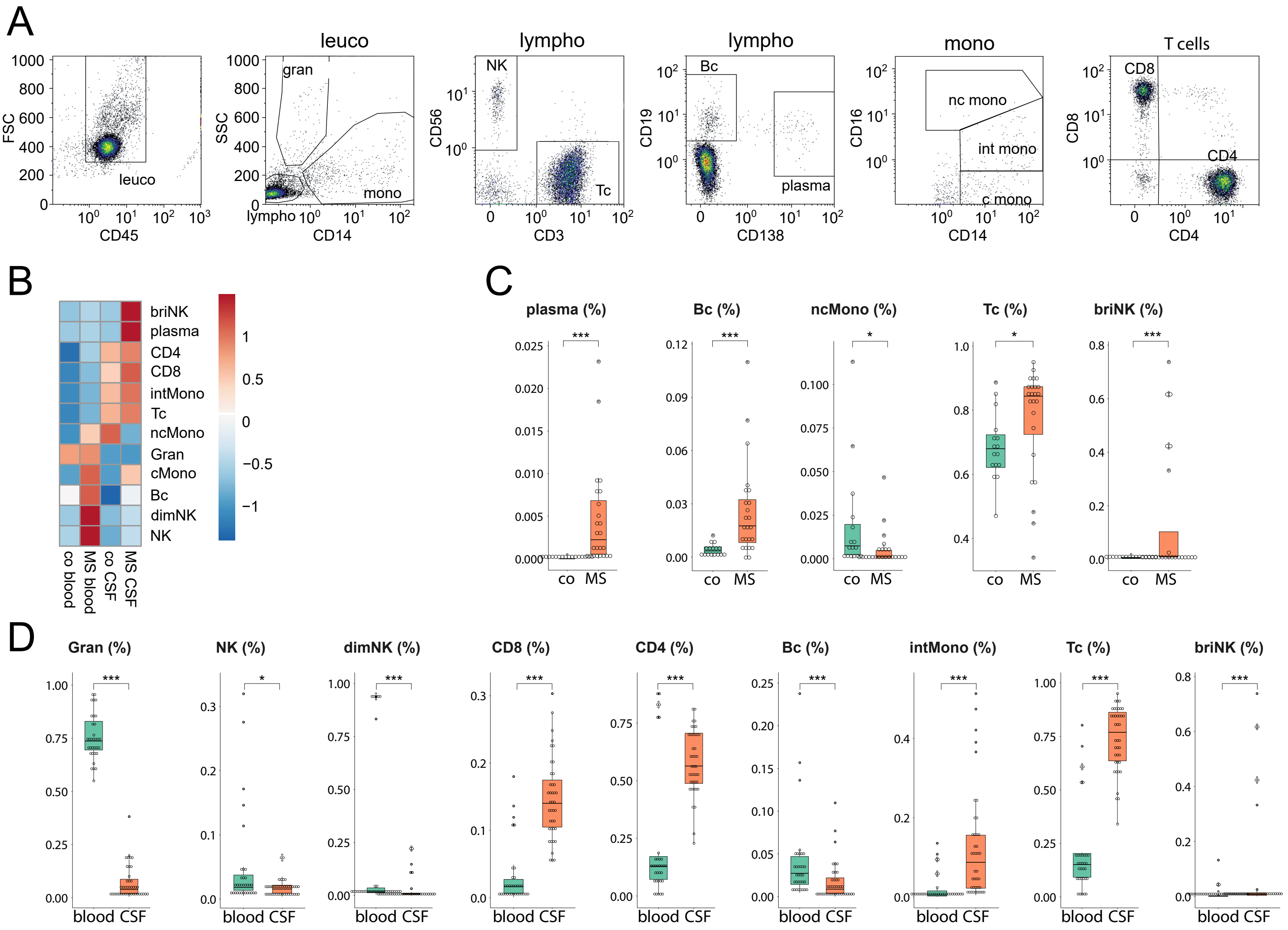

Supplementary Figure 4: Flow cytometry characterization of all CSF and blood samples.

(A) Representative gating strategy for identifying and quantifying cell types by flow cytometry in the CSF. Population names are indicated next to the respective gates. (B) Heatmap depicting the average proportion of cells in each population in control (co, n = 22) and multiple sclerosis (MS, n = 26) patients as quantified in all samples by flow cytometry. Heatmap color is scaled in each row to row average with color indicating higher (red) and lower (blue) than average. (C) CSF flow cytometry data are depicted as dot-boxplots if significantly (Mann-Whitney U test statistics) different between control and MS samples. Comparisons not depicted are not significantly different. Please note that none of the analyzed flow cytometry parameters was different between co and MS in blood. (D) Flow cytometry data are depicted as dot-boxplots if significantly different between blood and CSF. Mann-Whitney U test, *$P < 0.05$, **$P < 0.01$, ***$P < 0.005$. The lower and upper edges of the box plots represent the lower and the upper quartile, respectively, the horizontal line inside the box indicates the median, and the whiskers extend to the most extreme values within the 1.5 interquartile range of the lower/upper quartile. *leuco* leukocytes, *lympho* lymphocytes, *mono* monocytes, *Bc* B cells, *Tc* T cells, *CD4* $CD4^+$ T cells, *CD8* $CD8^+$ T cells, *dimNK / briNK* $CD56^{dim}$ / $CD56^{bright}$ natural killer cells, *plasma* plasma cells, *class mono / int mono / nc mono* classical / intermediate / non-classical monocytes, *gran* granulocyte. Source data for (B-D) listing the flow cytometry data are provided in the Source Data file.

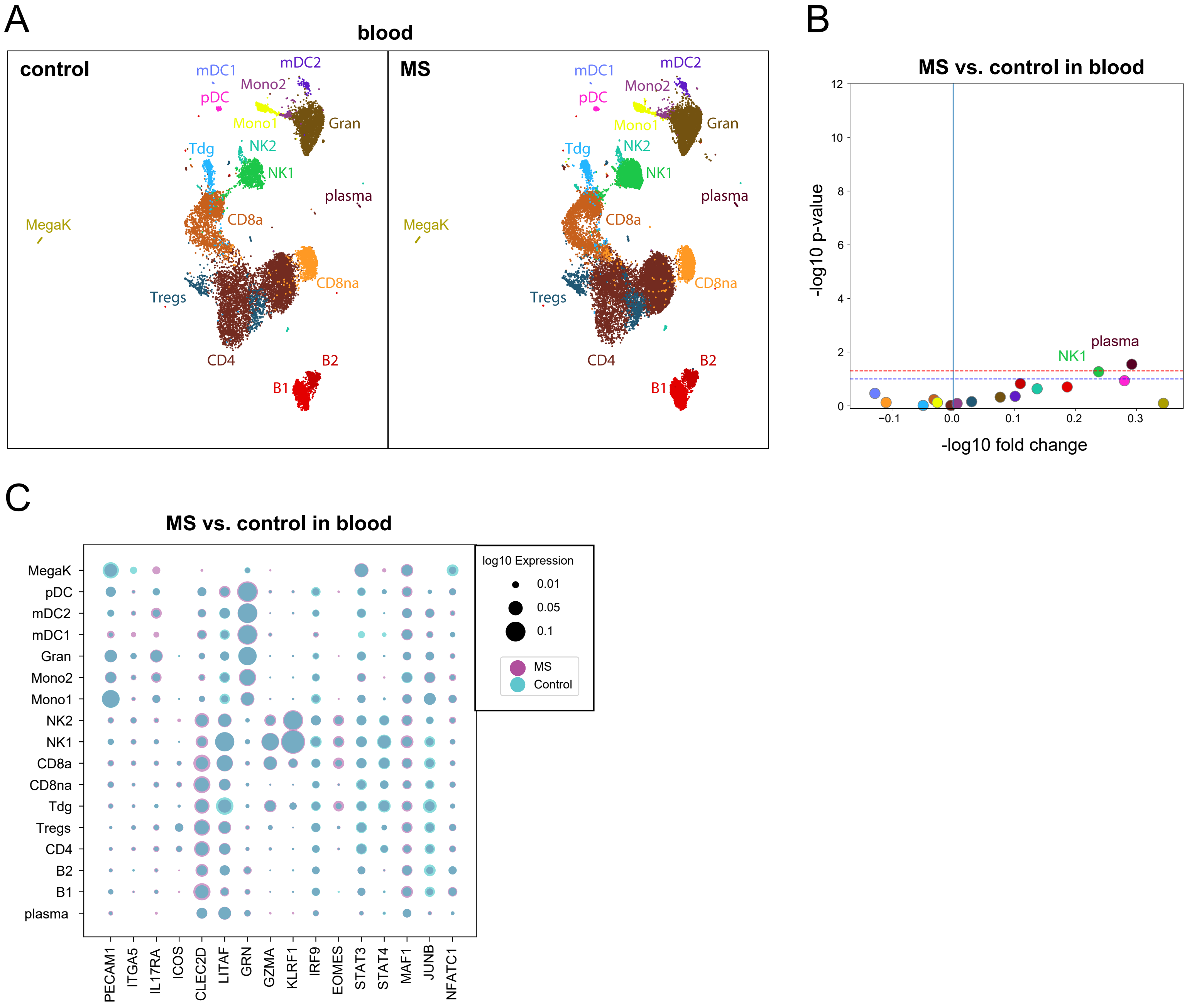

Supplementary Figure 5: Unlike CSF, multiple sclerosis does not affect cluster composition in blood.
(A) UMAP plot depicting all blood cell clusters separated by disease status from control (left) and multiple sclerosis (MS, right) patients. (B) Volcano plot depicting differences of cluster abundance among all blood cells in MS samples compared with control plotting fold change (log10) against p-value (-log10) based on binomial regression modeling (Methods). Horizontal line indicates significance threshold. (C) Dotplot depicting selected genes differentially expressed in some clusters of blood cells in MS compared to controls. Purple dot size encodes the average expression in MS and turquoise dot size encodes the average expression in controls. Dots are partially transparent thus overlap is dark, blue purple edge around dark blue circle indicates higher expression in MS, while turquoise edge around dark blue circle indicates lower expression in controls. Source data for (C) listing the differential expression values of MS vs. control in blood are provided in Supplementary Dataset 4.

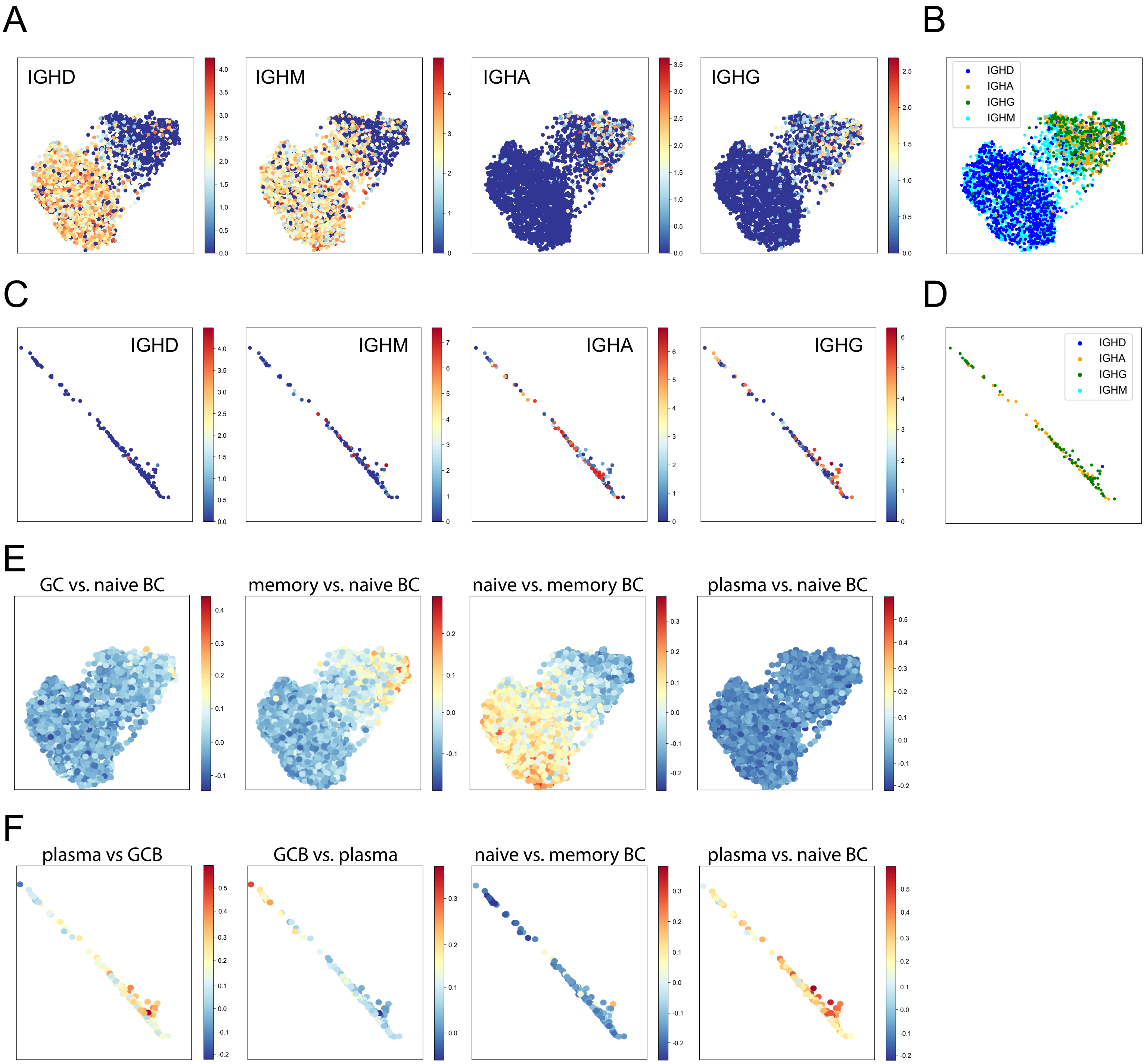

Supplementary Figure 6: Late B lineage cells accumulate in the CSF in MS.

(A) Feature plot showing the expression level of heavy chain genes IGHD/IgD, IGHM/IgM, IGHA/IgA, IGHG/IgG in the B cell clusters identified as B1/B2 in Figure 1A. Please note that IGHG summarizes IGHG1-4 genes and IGHA summarizes IGHA1-2 genes. (B) Cells expressing the respective IGH genes in the B cell clusters at maximum are highlighted. (C) Feature plots showing expression of heavy chain genes in the plasma cluster identified in Figure 1A. (D) Maximum expression of heavy chain genes in the plasma cluster. (E) UMAP feature plots for B cell clusters representing VISION signatures with significant VISION consistency scores ($P < 0.01$), from left to right: GC vs. naive B cells , memory vs. naive B cells, naive vs. memory B cells and plasma vs. naive B cells. (F) Feature plots for the plasma cell cluster representing VISION signatures with significant VISION consistency scores ($P < 0.01$), from left to right: plasma vs. GCB, GCB vs. plasma, naive vs. memory B cells and plasma vs. naive B cells. *GC* germinal center, *GCB* germinal center B cells.

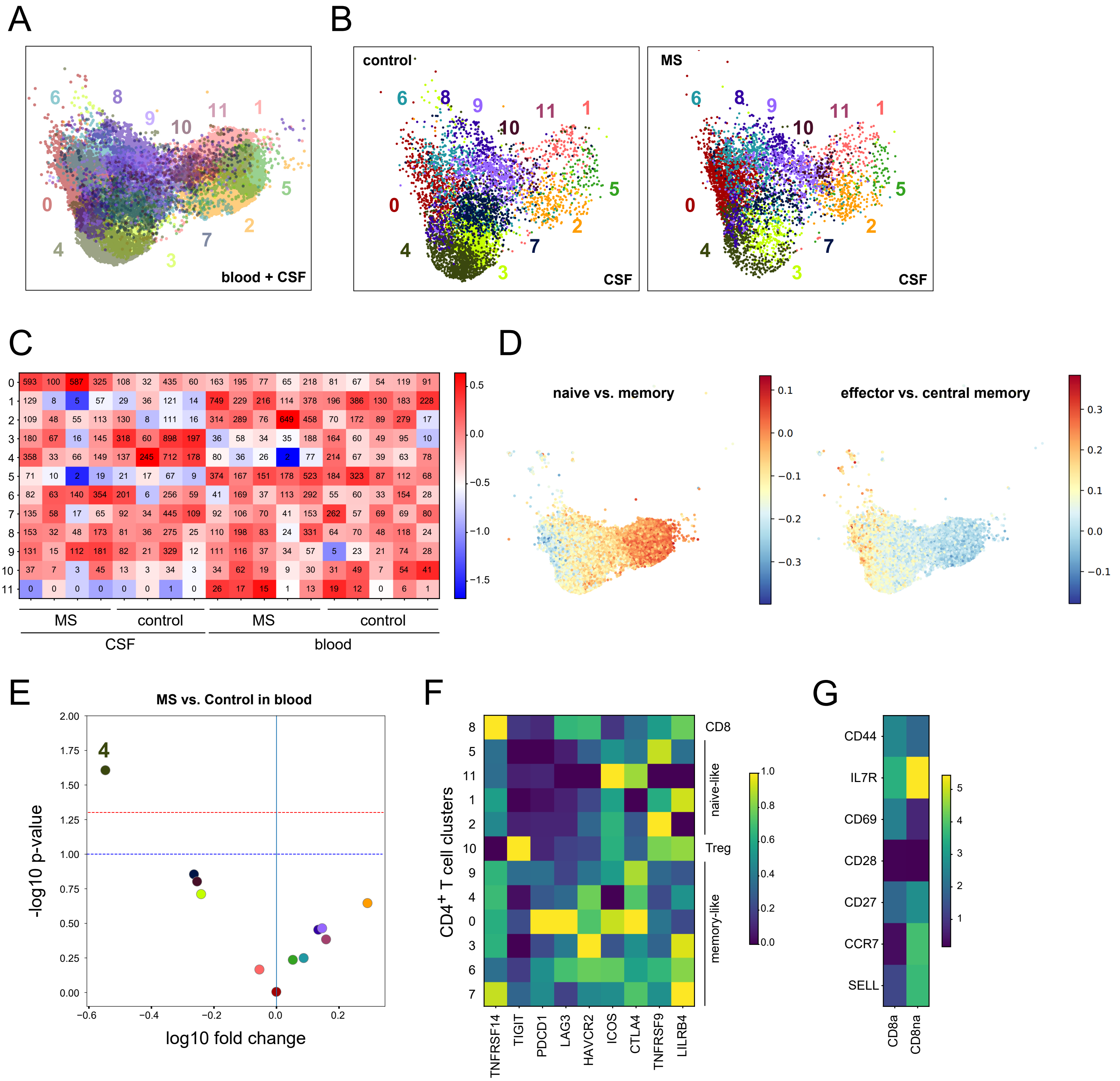

Supplementary Figure 7: $CD4^+$ T cells form a transcriptional continuum and show transcriptional rather than compositional changes in MS. (A) UMAP plot depicting sub-clustering of combined $CD4^+$ T cells from blood and CSF as in Figure 3A with opacity of every cell reduced to 50% to improve visibility of overlaid cells. Outlier cells were disregarded to improve resolution. (B) UMAP plot depicting $CD4^+$ T sub-clustered cells from CSF separated into control (left) and MS (right) cells. (C) Heatmap depicting cell numbers in each $CD4^+$ T sub-cluster sorted by tissue of origin and by disease-status. Numbers in the heatmap represent actual cell numbers. Color code indicates the relative abundance of cell types per donor, compared to the row average. (D) UMAP feature plots for $CD4^+$ T cells subclusters representing VISION signatures with significant VISION consistency scores ($P < 0.01$): (left) naïve vs. memory T Cell signature score[103], (right) late vs. early memory signature score[104]. (E) Volcano plot depicting differences of cluster abundance among all $CD4^+$ T cells from blood in MS samples compared with control plotting fold change (log10) against p-value (-log10) based on binomial regression modeling (Methods). (F) Heatmap showing average gene expression of selected exhaustion markers[46] in $CD4^+$ T cells combined from CSF and blood[50]. Expression values were normalized per gene with 0 reflecting the lowest expression and 1 reflecting the highest expression. (G) Heatmap showing expression of selected naïve (CCR7, SELL) vs. memory/activation markers (CD44, IL7R, CD69, CD28, CD27) in the two CD8 T cell populations. *CD8a* activated $CD8^+$ T cells, *CD8na* non-activated $CD8^+$ T cells. Source data for (F) listing the differential expression values of all $CD4^+$ T cells are provided in Supplementary Dataset 6. Source data for (G) listing the differential expression values of all $CD8^+$ T cells are provided in Supplementary Dataset 1.

A

blood

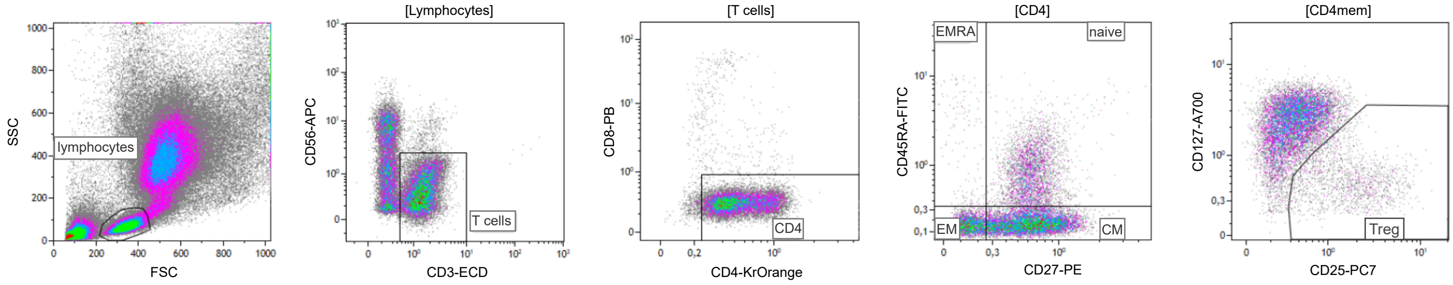

Supplementary Figure 8: Gating strategy for flow cytometry analysis of Treg and TEMRA cells.
(A) Representative gating strategy for identifying and quantifying Treg and TEMRA cell types by flow cytometry in the blood. Population names are indicated next to or within the respective gates. *T cells* CD3^{+}, *CD4* CD3^{+}CD4^{+}, *EM* effector memory CD3^{+}CD4^{+}CD45RA^{-}CD27^{-}, *EMRA* effector memory recently activated CD3^{+}CD4^{+}CD45RA^{+}CD27^{-}, *naive* CD3^{+}CD4^{+}CD45RA^{+}CD27^{+}, *CM* central memory CD3^{+}CD4^{+}CD45RA^{-}CD27^{-}, *Treg* CD3^{+}CD4^{+}CD127^{low}CD25^{high}.

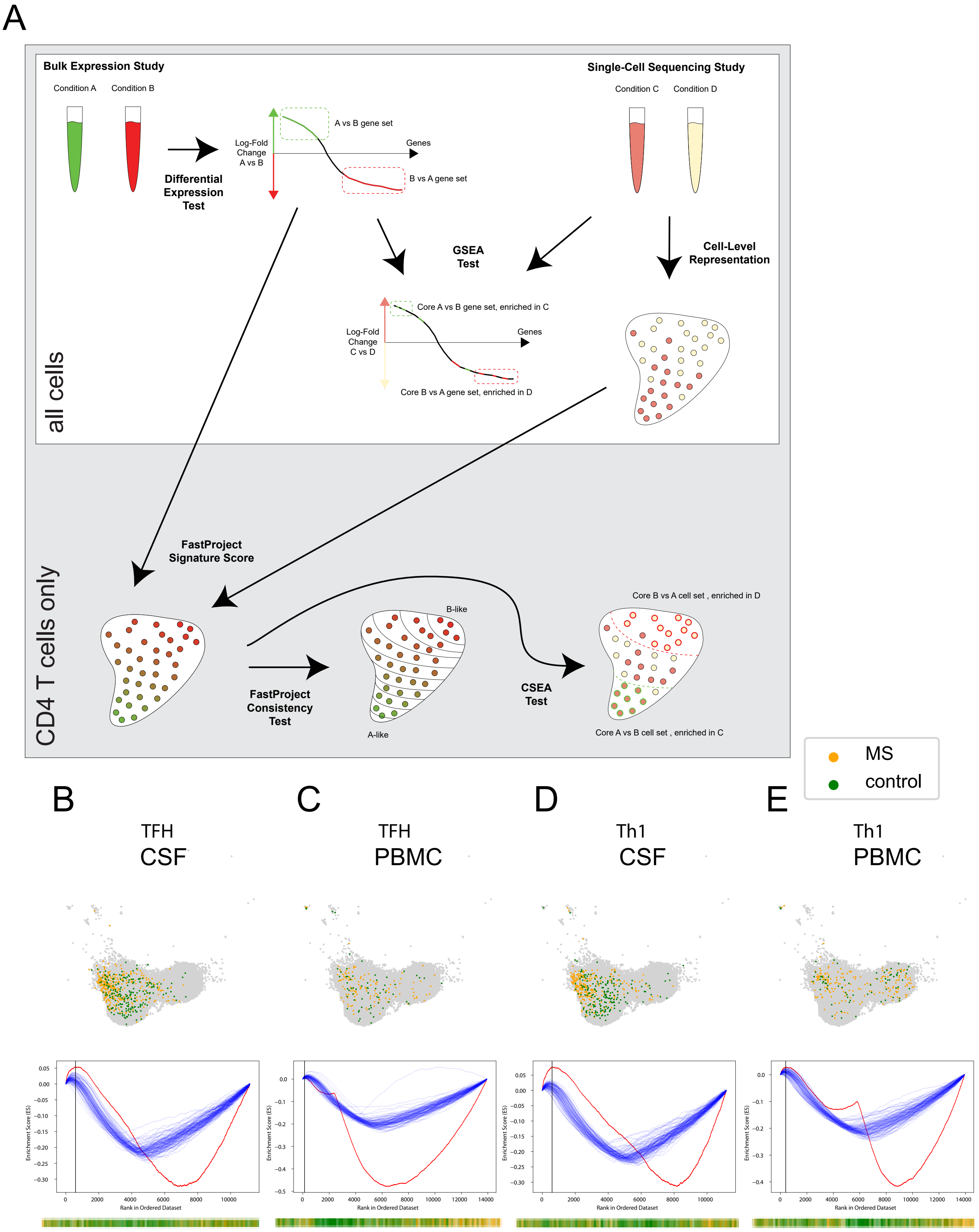

Supplementary Figure 9: Cell set enrichment analysis (CSEA) identifies cluster-independent transcriptional changes.
(A) Scheme of GSEA/VISION/CSEA Analysis. Available bulk expression data are used to identify gene signature sets characterizing immune cell populations (top left). These gene sets are used for either (i) gene set enrichment analysis (GSEA) of our scRNA-seq differential expression results (top middle) or (ii) single-cell VISION signature scores, input to both VISION consistency testing and cell set enrichment analysis (CSEA) testing (bottom). See Methods section for details. The red line is the enrichment score (ES) trajectory of the signature gene set, while the blue lines are the ES trajectories of all randomized genesets. A more extreme positive value in the left-most part of the figure indicates enrichment of cells expressing the signature set, compared to cells expressing the randomized genesets. (B-E) Two selected CSEA analysis using TFH marker gene set (B,C) and Th1 marker gene set (D,E) in the $CD4^+$ T cell analysis. TFH cells are significantly enriched in MS cells in CSF (B), but not in blood (C), while Th1 cells are enriched in both (D,E). Top row shows UMAP plots highlighting the leading edge cell sets from MS (orange) and control (green) samples. Cells depicted in grey are not part of the leading edge cell set of the respective signatures. Bottom row shows the enrichment score (y-axis) as a function of the rank of the cells by their Vision signature score (x-axis). The red line indicate the position of the leading edge. The 1D density plot shows the Vision signature score data with all MS cells in orange and control cells in green with the x-axis being the rank of the cells by their Vision signature score. Source data for (B-E) listing the CSEA results are provided in Supplementary Dataset 9.

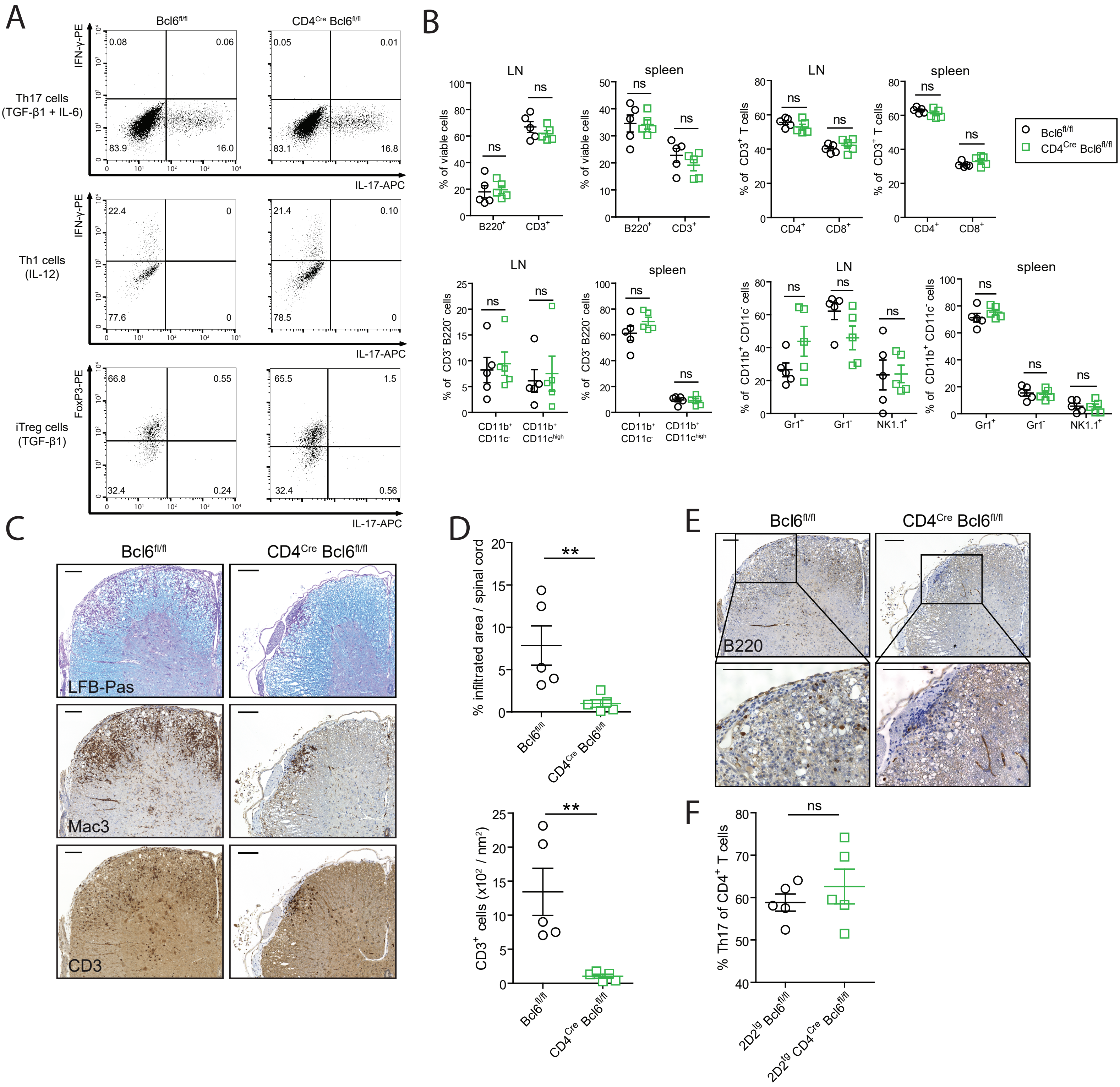

Supplementary Figure 10: Bcl6 deficiency in T cells does not grossly affect T helper cell differentiation and secondary lymphoid organs.
(A) Naïve $CD4^{+}CD62L^{high}CD44^{low}$ T cells were sorted from $Bcl6^{fl/fl}$ mice and $CD4^{Cre}Bcl6^{fl/fl}$ mice, differentiated in the presence of TGF-β1 and IL-6, or IL-12 alone, or TGF-β1 alone and analysed by intracellular cytokine staining after 4 days in culture. Gating strategy is depicted in Suppl. Fig. 16C. (B) At day 7 after active EAE induction, total viable cells from spleen and draining lymph nodes (LN) from $Bcl6^{fl/fl}$ mice and $CD4^{Cre}Bcl6^{fl/fl}$ mice were stained for CD3, B220, CD4, CD8, CD11b, CD11c, Gr1 and NK1.1. Gating strategy is depicted in Suppl. Fig. 16D. (C) At day 28 after active EAE induction, spinal cord paraffin cross-sections were stained for LFB-Pas, Mac3 and CD3. (D) The infiltrated area (top) and number of $CD3^{+}$ cells (bottom) per spinal cord was quantified manually in a blinded fashion. (E) Cross-sections of paraffin embedded spinal cords of $Bcl6^{fl/fl}$ (left) mice and $CD4^{Cre}Bcl6^{fl/fl}$ (right) mice were stained for B220. Scale bars represent 100 µm in panels C and E. (F) Naïve $CD4^{+}CD62L^{high}CD44^{low}$ T cells were sorted from $2D2tgBcl6^{fl/fl}$ and $2D2tgCD4^{Cre}Bcl6^{fl/fl}$ donor mice and differentiated into pathogenic Th17 cells (Methods). After 5 days of culture, cells were analysed by intracellular cytokine staining. One representative out of five independent experiments is shown. Sorting strategy is depicted in Suppl. Fig. 16C. Mann-Whitney U test, $^{*}P < 0.05$, $^{**}P < 0.01$, *ns* not significant. Dotplots show mean ± SEM, Source data for (B,D,F) are provided in the Source Data file.

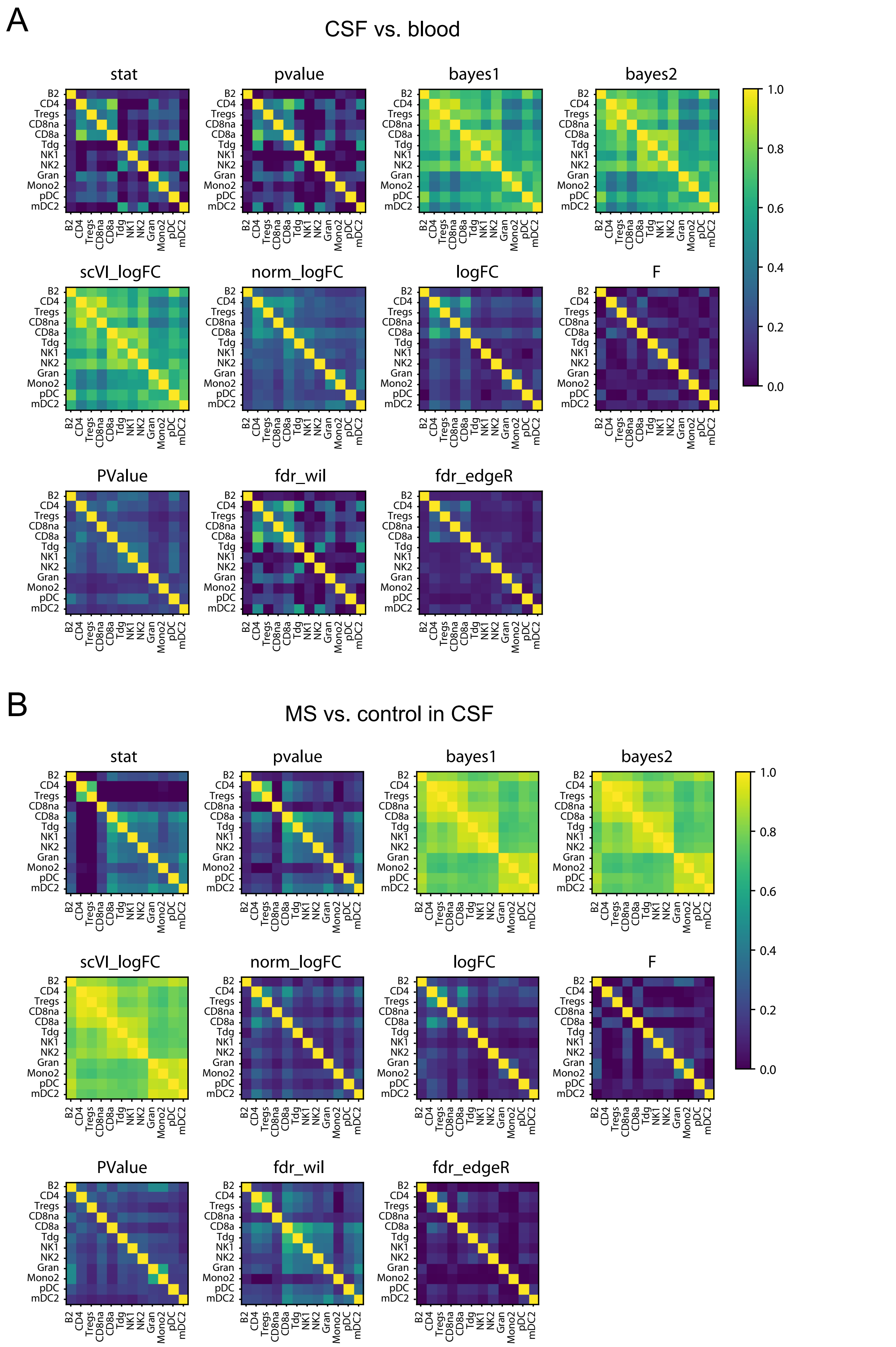

Supplementary Figure 11: Correlation between different measures of differential expression.
(A) Matrix of Spearman rank correlation r of the different measures of differential expression of individual genes within clusters in the CSF vs. blood comparison. For example, the Bayes1 factor of all genes in the CD4 cluster is more closely correlated (i.e. yellow) with all genes in the CD8na cluster than with all genes in the Gran cluster. (B) Matrix of Spearman rank correlation r of the different measures of differential expression of individual genes within clusters in the MS vs. control comparison within CSF. Source data for (A) listing the differential expression values of CSF vs. blood are provided in Supplementary Dataset 2. Source data for (B) listing the differential expression values of MS vs. control in CSF are provided in Supplementary Dataset 5.

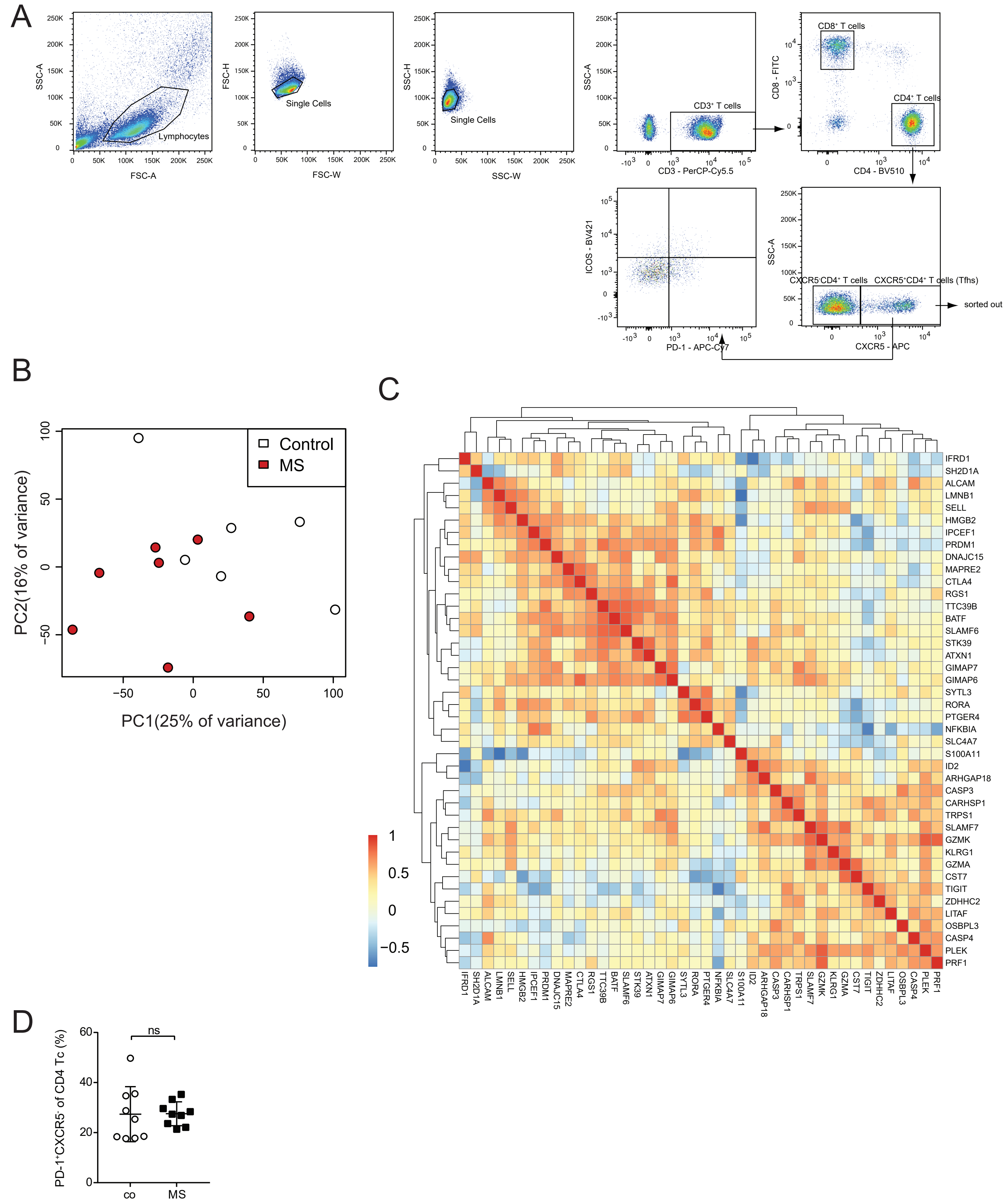

Supplementary Figure 12: TFH sorting, characterisation and RNA bulk-seq.

(A) Representative gating strategy for the analysis and sorting of human Tfh cells from CSF and blood. (B) $CD3^+CD4^+CXCR5^+$ (Tfh) cells quantified in Figure 4B were flow sorted from the CSF of control patients (n = 9, 237±107 SD cells) and MS patients (n = 9, 852±691 SD cells), followed by RNA-seq that was technically successful in 6 control vs. 7 MS patients, respectively. Scatter plot depicts a principal component analysis (PCA) of normalized TFH gene log-count data color-coded by disease status. (C) No individual genes reached the significance threshold after correction for multiple hypothesis testing. Genes were sorted by the significance score obtained in the MS vs. control comparison were input into GSEA to identify enrichments. The most frequent members of core gene sets driving enrichment in MS are plotted in a gene-gene Pearson correlation matrix (Methods). (D) CSF from control vs. MS patients was analyzed for the presence of $PD\text{-}1^+CXCR5^-$ CD4 T cells. Mann-Whitney U test, *ns* not significant. Dotplots show mean ± SD, Source data for (C) listing the differential expression values and GSEA results are provided in Supplementary Dataset 10. Source data for (D) are provided in the Source data file.

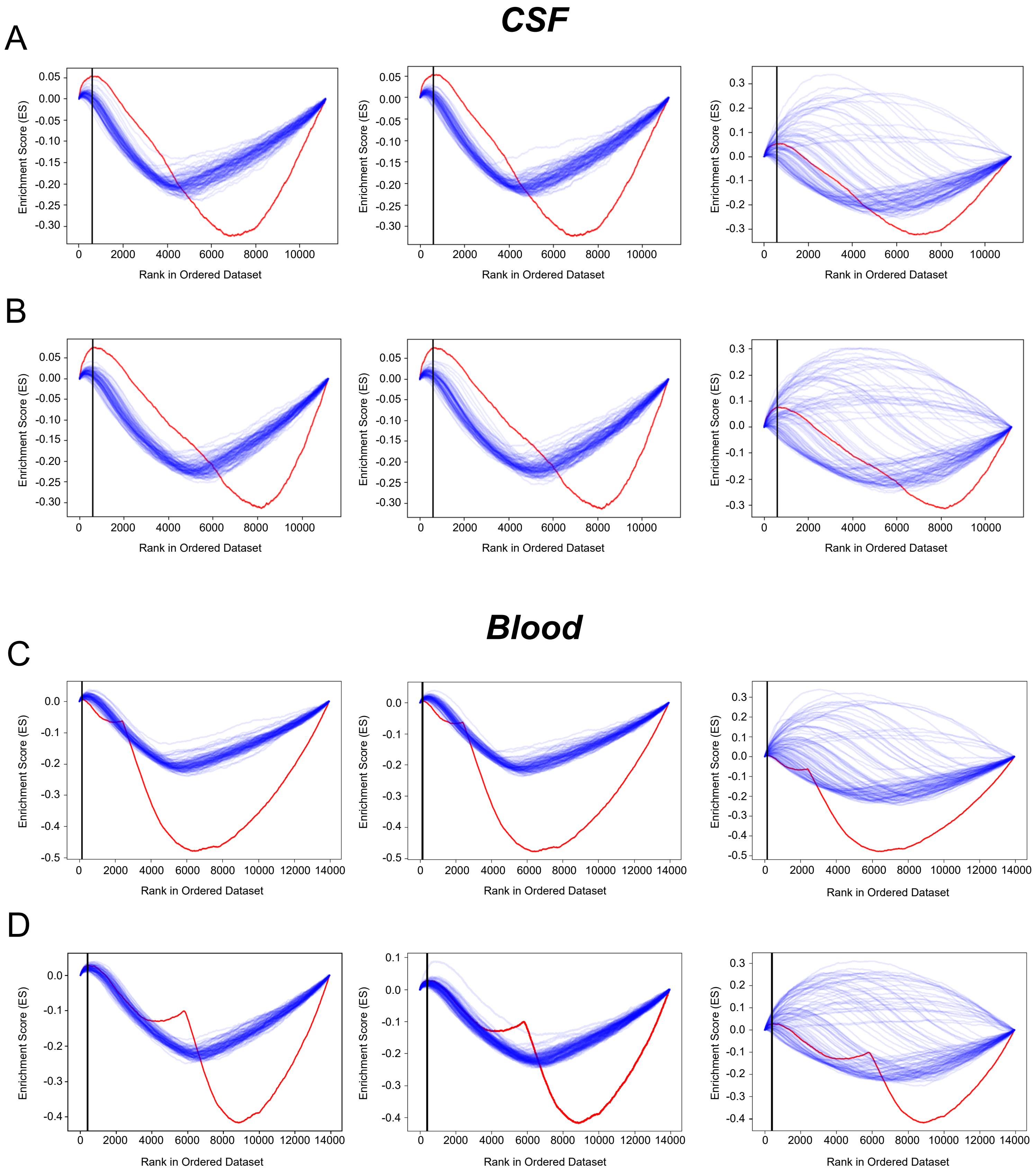

Supplementary Figure 13: Testing CSEA against genesets with lower matching.
The performance of CSEA was tested using two less stringently matched randomized genesets (left and middle panels), and one randomized geneset that is sampled from all available genes (right panel). Compare results to Supplementary Figure 9. Results of the TFH (A) and the Th1 (B) signature in CSF are depicted and results of the TFH (C) and the Th1 (D) signatures in blood are depicted. The red line is the enrichment score (ES) trajectory of the signature gene set, while the blue lines are the ES trajectories of all randomized genesets. A higher positive value in the left-most part of the figure indicates enrichment for cells expressing the signature set, compared to cells expressing the randomized genesets. The randomized genesets were selected from the top 100 average-expression-matched genes (left panels), from 500 matched genes (middle panels), or from genes that were sampled completely randomly

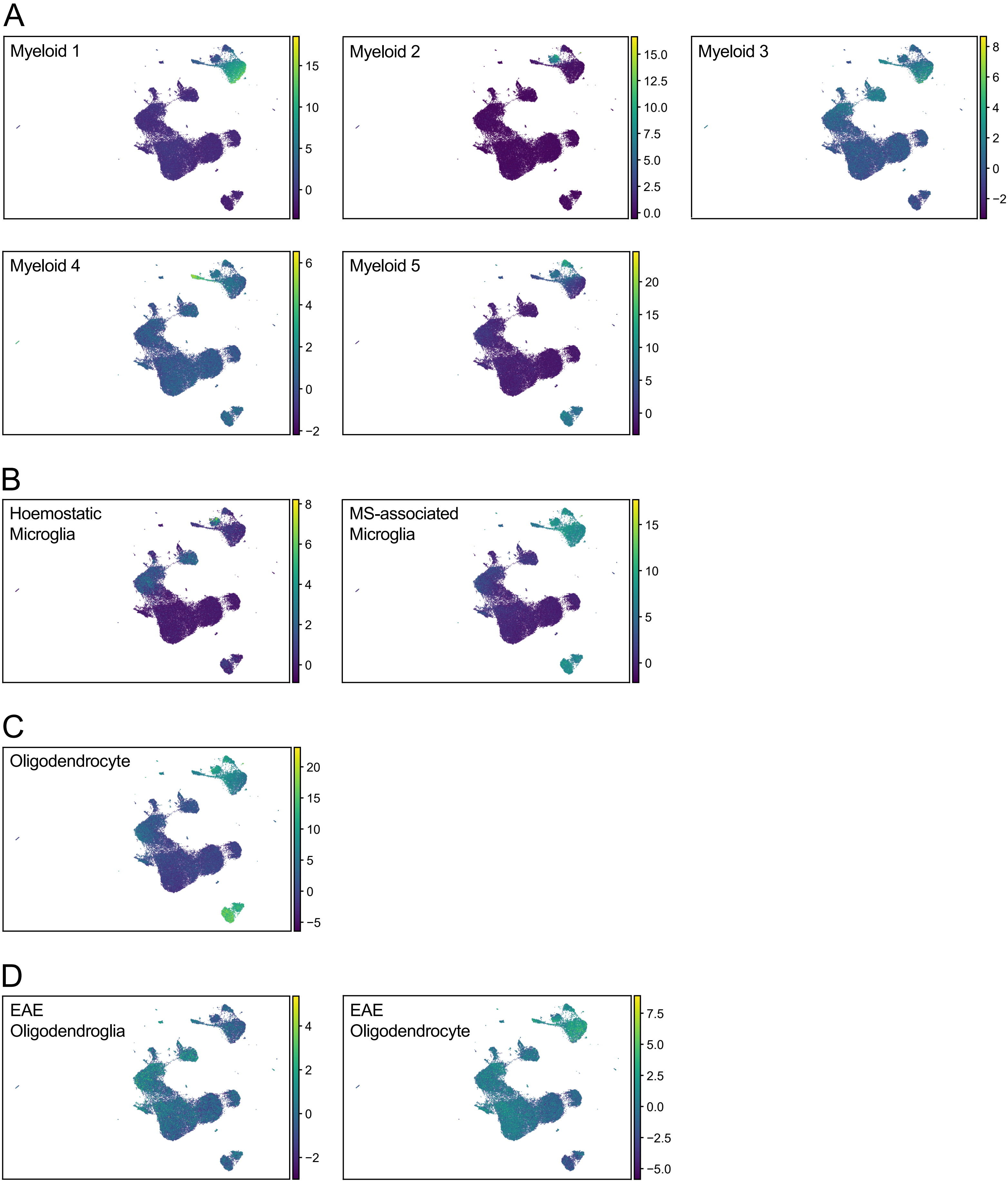

Supplementary Figure 14: Comparison with published gene signature sets.

We obtained the key marker genes of myeloid lineage cell clusters from recent publications[8,34,89,90]. The combined expression level of the respective gene signatures was plotted onto the combined blood and CSF dataset. Brighter, yellow color indicates that higher expression of a certain signature set. Gene sets were derived in (A) from reference[8], in (B) from reference[34], in (C) from reference[89], and in (D) from reference[90].

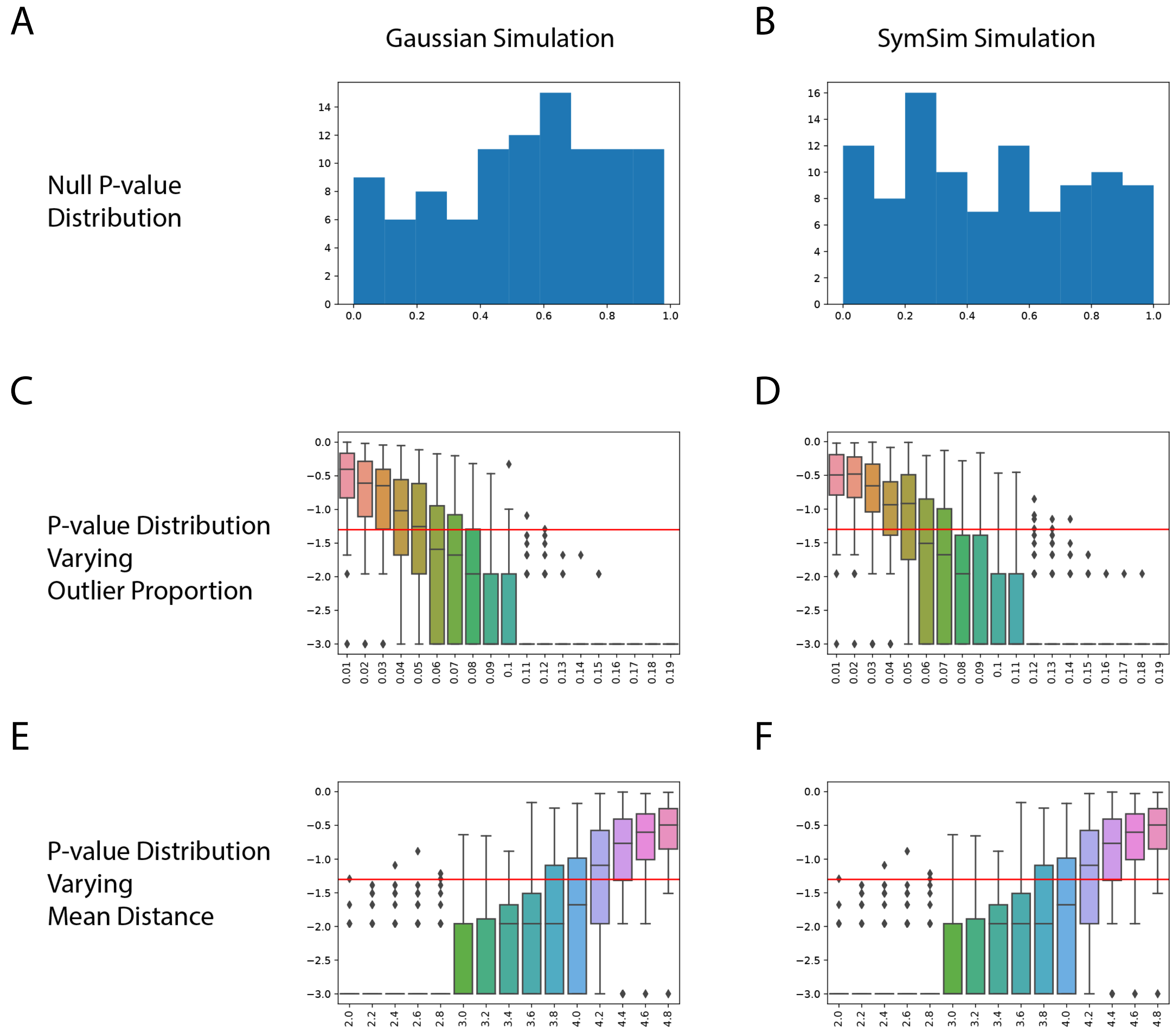

Supplementary Figure 15: CSEA validation.

The distribution of P-value from CSEA is shown in this figure. The first column corresponds to the simple, distribution-based simulation using mixtures of Guassian distributions, and the second column corresponds to the more realistic, gene-expression-based simulation using SymSim. (A,B) Distribution of P-value when there is no differences between the two samples, and the P-values are expected to be uniformly distributed. The x-axis shows the P-value, and the y-axis shows the counts in each bin with 100 replicates. (C,D) The boxplots show the distribution of P-value for 10 replicates for each outlier proportion. The two distributions have an identical portion and one of the two have an outlier population. As the proportion of outlier increases, the power to detect the difference also increases, as reflected by the decrease in log P-value. The red line indicates the significance threshold of 0.05. The mean difference between the two populations of cells is fixed at 2 standard deviations. (E,F) The boxplots show the distribution of P-value for 10 replicates for each mean differences. The two distributions have an identical portion and one of the two have an outlier population that consists of 10% of all cells. As the mean of the outlier moves farther away from the mean of the main distribution (fixed at 5), the power to detect the difference also increases, as reflected by the decrease in log P-value. The red line indicates the significance threshold of 0.05. The lower and upper edges of the box plots represent the lower and the upper quartile, respectively, the horizontal line inside the box indicates the median, and the whiskers extend to the most extreme values within the 1.5 interquartile range of the lower/upper quartile.

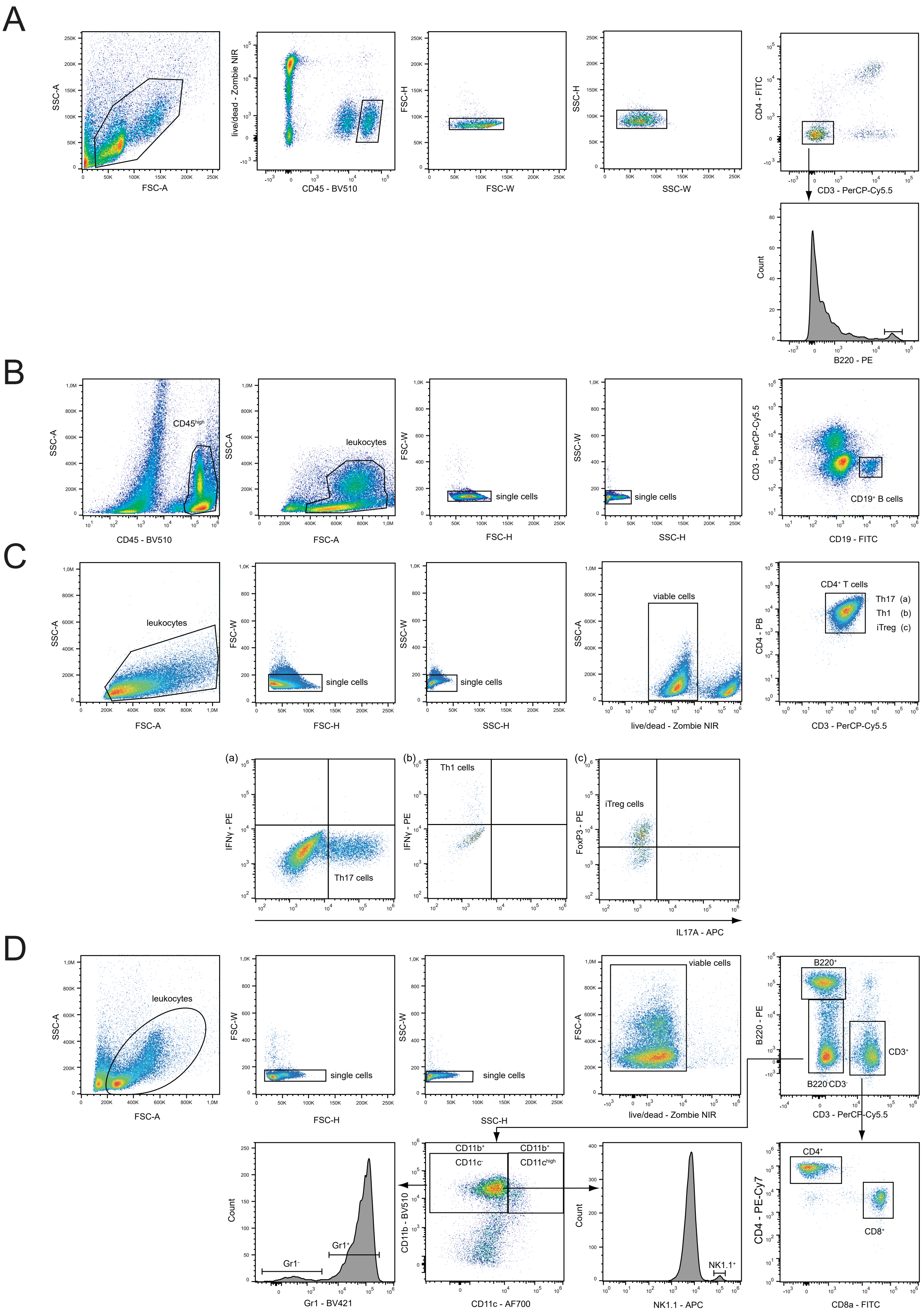

Supplementary Figure 16: Representative gating strategies for murine flow cytometry characterisations.

(A) Representative gating strategy for the B cell characterisation in Bcl6$^{fl/fl}$ and CD4^{Cre}Bcl6$^{fl/fl}$ mice as shown in Fig. 4D.

(B) Representative gating strategy for the B cell characterisation in 2D2^{tg}Bcl6$^{fl/fl}$ and 2D2^{tg}CD4^{Cre}Bcl6$^{fl/fl}$ mice as shown in Fig. 4F.

(C) Representative gating strategy for the analysis of *in vitro* differentiated Th17/Th1/iTreg cells as shown in Suppl. Fig. 10A,F.

(D) Representative gating strategy for the characterization of the cell composition in secondary lymphoid organs from Bcl6$^{fl/fl}$ and CD4^{Cre}Bcl6$^{fl/fl}$ mice as shown in Suppl. Fig.10B.

	name of cohort	Included	average age (±SD)	Female	Male	Excluded
Total		66	34.1 ± 9.0	50	16	48
IIH total		27	35.2 ± 8.4	20	7	27
IIH cohort 1	scRNAseq	6	33.4 ± 8.1	4	2	
IIH cohort 2	flow only	7	35.1 ± 9.3	4	3	
IIH cohort 3	TFH RNAseq	9	34.2 ± 8.8	8	1	
IIH cohort 4	validation	5	39.4 ± 8.0	4	1	
MS total		39	33.7± 9.5	30	9	21
MS cohort 1	scRNAseq	6	36.7 ± 10.3	4	2	
MS cohort 2	flow only	12	32.2 ± 12.4	9	3	
MS cohort 3	TFH RNAseq	9	33.1 ± 6.8	8	1	
MS cohort 4	validation	12	34.1 ± 8.2	9	3	

Supplementary Table 1: Summarized information of patients in the present study.

Summarized characteristics of patients included in the present study. Patients were divided into four cohorts; cohort 1: CSF and blood samples used for single cell RNA-seq. (6 control vs. 6 MS), cohort 2: CSF and blood samples analysed by flow cytometry only (7 control vs. 12 MS) and cohort 3: CSF samples flow sorted for RNA-seq of $CD3^{+}CD4^{+}CXCR5^{+}$ TFH cells (9 control vs. 9 MS), cohort 4: CSF samples analysed by flow cytometry for the proportion of $CD4^{+}$ T effector memory cells recently activated (TEMRA cells) and regulatory T cells (5 control vs. 12 MS).

pseudonym	group	cohort	age	sex	#stFLAIR les	MRI DIS+	Gd+	OCB DIT+	clinical DIT+	#prior rel	EDSS @DC	latest FU	CDMS @FU	EDSS @FU	cell	lympho	granulo	rbc	protein	lactate	glucose	OCB+	OCB type	CSF index
44512	co	flow only	50	f											NA	NA	NA	NA	NA	NA	NA	NA	NA	NA
49410	co	flow only	39	f											1	1	0	79	564	1.84	70	NA	NA	NA
50574	co	flow only	23	m											4	4	0	0	485	1.31	62.5	NA	NA	barrier only
17340	co	flow only	26	f											1	1	0	0	351	NA	NA	NA	NA	NA
99739	co	flow only	43	m											6	6	0	0	873	1.9	102	NA	NA	NA
19645	co	flow only	32	f											1	1	0	0	766	1.92	82.8	0	I	barrier only
73252	co	flow only	33	m											1	1	0	4	480	1.79	79.2	0	I	none
95809	co	scRNA-seq	43	f											1	NA	NA	NA	199	NA	NA	NA	NA	NA
83775	co	scRNA-seq	43	m											4	4	0	0	796	1.3	81.7	0	I	barrier only
41540	co	scRNA-seq	32	f											2	2	0	18	329	1.38	67	NA	NA	none
85037	co	scRNA-seq	25	f											1	1	0	5	317	1.46	55.4	0	I	none
32190	co	scRNA-seq	33	m											1	1	0	0	447	1.66	81.7	0	I	none
45044	co	scRNA-seq	25	f											1	NA	NA	NA	244	1.64	62.4	NA	I	none
13697	co	TFH	40	f											0	0	0	0	140	1.72	54.6	0	I	none
91898	co	TFH	23	f											1	1	0	0	753	1.55	61.6	0	I	barrier only
65767	co	TFH	31	f											1	1	0	0	311	NA	NA	NA	NA	none
12400	co	TFH	37	f											2	2	0	0	397	1.88	80.3	0	I	none
48865	co	TFH	53	m											0	0	0	0	495	2.35	115	0	I	none
14713	co	TFH	34	f											1	1	0	0	376	1.65	58	0	I	none
78572	co	TFH	27	f											0	0	0	0	364	1.79	72.6	0	I	none
65946	co	TFH	37	f											0	0	0	0	578	1.54	79.3	0	I	barrier only
86323	co	TFH	28	f											2	2	0	0	436	1.17	55.9	0	I	barrier only
88490	co	validate	34	f											0	0	0	0	380	1.47	71	0	I	none
74229	co	validate	39	f											8	8	0	8	323	1.83	79.1	1	II	Ig only
55421	co	validate	38	f											0	0	0	0	565	1.43	54.6	0	I	barrier only
68576	co	validate	53	m											0	0	0	0	352	0.72	103	0	I	none
80034	co	validate	33	f											0	0	0	0	190	1.66	68.1	0	I	none
55597	MS	flow only	56	f	>10	1	1	1	1	1	2.0			2.0	12	12	0	0	793	4.6	165	1	II	barrier and Ig
85397	MS	flow only	24	m	6	1	0	1	0	0	1.5			1.5	2	2	0	0	255	2.39	91	1	II	none
94258	MS	flow only	21	f	8	1	1	0	1	2	1.0			1.5	0	0	0	0	722	1.62	44	0	I	barrier only
73909	MS	flow only	23	f	>10	1	0	1	1	3	1.0			1.0	8	8	0	0	367	1.57	60.4	1	III	Ig only
90707	MS	flow only	19	f	>10	1	1	0	0	0	1.0	22	1	1.5	2	2	0	0	285	1.65	57	0	I	none
47457	MS	flow only	20	f	>10	1	1	1	1	1	2.0			2.0	2	2	0	0	377	2.36	143	1	II	none
48203	MS	flow only	28	f	>10	1	1	1	1	1	1.5			1.5	4	4	0	0	672	2.06	75.5	1	II	Ig only
12597	MS	flow only	42	m	>10	1	1	1	0	0	0.0	9	1	0.0	58	58	0	0	618	1.9	87.1	1	III	none
90414	MS	flow only	24	f	>10	1	0	1	1	2	0.0			0.0	10	10	0	0	365	1.66	58.6	1	II	Ig only
22577	MS	flow only	44	f	6	1	0	1	0	0	1.0	19	1	1.5	1	1	0	0	318	1.83	53.4	1	III	Ig only
14096	MS	flow only	42	f	>10	1	1	1	0	0	1.0	12	1	1.0	11	11	0	0	431	1.99	59.8	1	III	Ig only
19774	MS	flow only	44	m	>10	1	1	1	1	2	3.5			3.0	13	13	0	192	644	1.83	69.2	1	II	barrier only
60249	MS	scRNA-seq	28	f	>10	1	1	1	1	3	1.0			1.0	22	22	0	0	678	1.7	67	1	II	barrier and Ig
58637	MS	scRNA-seq	22	m	7	1	1	1	0	0	0.0	6	1	0.0	5	5	0	0	429	1.9	54	1	II	Ig only
74594	MS	scRNA-seq	42	m	5	1	0	0	1	1	0.0			0.0	NA	NA	NA	NA	NA	NA	NA	NA	NA	NA
19270	MS	scRNA-seq	35	f	8	1	0	1	0	1	0.0	6	1	0.5	3	3	0	0	410	2.1	75	1	II	Ig only
71658	MS	scRNA-seq	47	f	>10	1	1	1	1	2	6.0			6.0	0	0	0	0	368	1.53	59	1	II	Ig only
49131	MS	scRNA-seq	47	f	6	1	1	1	1	1	4.5			2.5	0	0	0	2	521	1.96	69	1	III	Ig only
35648	MS	TFH	47	f	9	1	0	1	0	0	3.0	5	1	3.0	0	0	0	0	616	1.79	77.5	1	II	Ig only
41201	MS	TFH	25	f	4	1	0	0	0	0	1.0	8	1	1.0	1	1	0	0	259	2.51	106	0	I	none
66914	MS	TFH	33	f	5	1	1	1	0	0	0.5	6	1	0.5	17	17	0	0	474	2.23	90.6	1	II	none
52035	MS	TFH	32	f	2	0	1	0	0	0	1.0	5	1	1.0	4	4	0	0	594	1.48	64.6	0	I	none
62103	MS	TFH	28	m	9	1	1	1	1	1	1.5			0.0	18	18	0	108	627	1.8	69	1	II	Ig only
84636	MS	TFH	33	f	3	0	1	0	0	0	0.5	5	1	1.0	5	5	0	2	442	2.46	111	0	I	none
44212	MS	TFH	31	f	4	1	1	1	1	2	1.0			1.0	9	9	0	0	255	1.6	60	1	II	Ig only
85578	MS	TFH	40	f	6	1	1	1	1	1	1.0			1.5	2	2	0	0	285	2.05	74.7	1	II	Ig only
60406	MS	TFH	28	f	1	0	0	0	0	0	1.0	8	1	1.0	0	0	0	0	398	1.45	56.8	0	I	none
90180	MS	validate	49	f	>10	1	1	1	0	0	1.0	9	1	1.0	5	5	0	0	1050	1.95	66.3	1	III	barrier only
29249	MS	validate	23	f	>10	1	0	1	0	0	1.0	13	1	1.0	52	48	0	0	687	2.11	70.5	1	III	barrier and Ig
32736	MS	validate	34	f	9	1	0	1	1	3	2.0			3.0	9	9	0	0	214	1.73	64	1	II	Ig only
36293	MS	validate	40	m	>10	1	0	1	1	3	3.0			3.0	19	19	0	0	651	1.94	65.1	1	II	none
88744	MS	validate	30	m	4	1	0	1	1	1	2.0			0.0	10	10	0	0	576	1.91	92.2	1	II	Ig only
83197	MS	validate	32	f	>10	1	1	1	1	3	4.0			4.0	2	2	0	0	374	1.09	52.7	1	II	none
22249	MS	validate	20	f	>10	1	1	1	1	1	2.5			1.5	5	5	0	0	304	2.01	73.2	1	III	Ig only
97413	MS	validate	38	f	4	1	1	1	0	0	1.0	7	1	1.0	8	8	0	0	403	2.61	80.5	1	II	Ig only
95415	MS	validate	28	f	>10	1	1	0	1	2	3.0			3.0	2	0	0	0	412	1.63	61.8	0	I	none
11419	MS	validate	36	f	9	1	1	1	0	0	0.5	10	1	1.0	2	2	0	168	435	2.03	67.5	1	II	Ig only
41442	MS	validate	36	f	8	1	0	1	1	1	1.0			1.0	3	3	0	0	294	2.48	66.8	1	II	none
38519	MS	validate	43	m	>10	1	1	1	0	0	2.0	5	1	2.0	21	0	0	0	557	1.53	60.5	1	III	Ig only

Supplementary Table 2: Detailed features of patients in the present study.

MS disease and MRI features and CSF parameters of all control (IIH, n=27) and multiple sclerosis (MS, n=39) patients included in the present study are listed. All included patients were divided into four cohorts. See Methods and Suppl. Tab. 1 for details regarding the cohort naming. Column legend: co control patient, MS multiple sclerosis patient, f female, m male, #stFLAIR les number of supratentorial FLAIR hyperintense lesions in magnetic resonance imaging (MRI), MRI DIS+ criteria for dissemination in space (DIS) fulfilled by MRI at time of lumbar puncture (LP), Gd+ any gadolinium enhancing lesion detected in MRI, OCB DIT+ criteria for dissemination in time (DIT) fulfilled by the presence of oligoclonal bands (OCB) in CSF at time of LP, clinical DIT+ criteria for dissemination in time fulfilled by the presence of at least two clinical relapses, #prior rel number of clinical relapses prior to the one occurring at LP, EDSS @DC Kurtzke Expanded Disability Status Scale (EDSS) score at discharge i.e. after treatment of the relapse occurring at LP, latest FU latest available clinical follow-up in months after LP, CDMS @FU clinically defined relapsing remitting (RR)MS at latest clinical follow-up, i.e. documented occurrence of at least one additional relapse after the relapse occurring at LP, EDSS @FU EDSS score at latest clinical follow-up, cell total cells per µl, lympho lymphocytes per µl, granulo granulocytes per µl, rbc red blood cells per µl, protein protein concentration in CSF in mg/l, lactate lactate concentration in CSF in nmol/l, glucose glucose concentration in CSF in mg/dl, OCB+ any oligoclonal band (OCB) detected in CSF, OCB type OCB were classified as being either undetectable (type 1), restricted to CSF (type 2), detected in serum and additionally in CSF (type 3), or not determined (NA), CSF index CSF/serum indices for albumin and immunoglobulin G (IgG) were calculated. The CSF barrier function (CSF index) was evaluated as being either unaffected (none), showing intrathecal IgG synthesis (Ig only), showing barrier dysfunction (barrier only), or showing both intrathecal IgG synthesis and barrier dysfunction (barrier and Ig).

Please note that patient 71658 had a severe first relapse with poor recovery, but no secondary progressive MS. Please note that patients 52035, 84636, 60406 did not fulfill criteria for DIS by MRI at time of LP, but were confirmed to have developed clinically defined RRMS at follow-up. Please note that all CSF analyses including OCB analysis failed technically in patient 74594 at the time of LP. This patient had OCB type II in an LP performed during a later clinical relapse.

	number of samples included	total number of measured cells	average number of measured cells (± SEM)	reads per cell (± SEM)	genes per cell (± SEM)
CSF_control	4	12705	3176.3 ± 2811.3	3774.80 ± 1958.80	1026.11 ± 374.84
CSF_MS	4	9652	2413 ± 1038.1	3367.58 ± 2241.05	1015.09 ± 372.81
blood_control	5	17138	3427.6 ± 1476.9	3376.29 ± 1685.92	808.76 ± 389.29
blood_MS	5	25831	5166.2 ± 1701.3	3438.51 ± 1811.71	1017.73 ± 347.97

Supplementary Table 3: Technical information of scRNA-seq results.
Technical information on scRNA-seq results of all patients (Control, n=4 and MS, n=4) included in the study are listed. Depicted is the number of samples used for scRNA-seq (number of samples for 10x), the total number of expected cells based on counting cells included in each sample multiplied by the approximate capture rate of the 10x system (total number of expected cells), the total number of measured cells after sequencing and genome alignment (total number of measured cells), average number of measured cells per sample (average number of measured cells), average number of detected reads per cell (reads per cell) and average number of detected genes per cell (genes per cell) used for downstream analysis. The total and average number of cells measured within the $CD4^{+}$ T cell (CD4_Tc) cluster is also depicted.
