# Supplementary material for: Integrated single cell analysis of blood and cerebrospinal fluid leukocytes in multiple sclerosis
Source: Nat Commun. 2020 Jan 14;11:247. doi: 10.1038/s41467-019-14118-w (PMC6959356; doi:10.1038/s41467-019-14118-w)

Cell Set Enrichment Analysis (CSEA)

In [1]:
```
import numpy as np
import scipy
from scipy.stats import poisson, uniform
import seaborn as sns
import matplotlib.pyplot as plt
import gseapy
import pandas as pd
import random
import copy
import numpy.random
import warnings
warnings.simplefilter('ignore')
```

In [2]:
```
np.random.seed(52)
```

Generating a simple distribution of normal and diseased cells using a beta distribution

In [3]:
```
def gen_distribution(is_normal):
    if is_normal:
        data_normal = np.random.beta(2.3, 5, 1000)
        return np.sort(data_normal)
    else:
        data_diseased = np.random.beta(2, 5, 1000)
        return np.sort(data_diseased)
```

In [4]:
```
diseased_cell = gen_distribution(0)
```

In [5]:
```
normal_cell = gen_distribution(1)
```

In [6]:
```
plt.hist(normal_cell, color='green', label='normal')
plt.hist(diseased_cell, color='orange', label='diseased')
plt.xlabel("Cell Stage")
plt.ylabel("Frequency")
plt.legend()
plt.show()
```

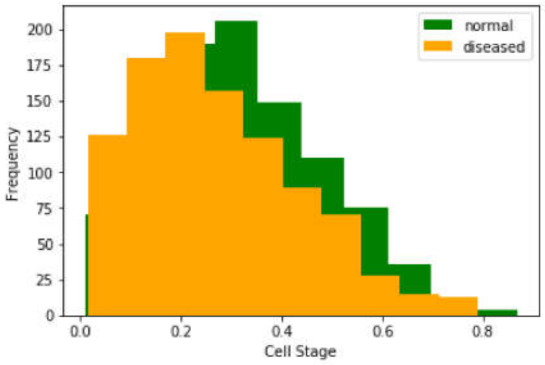

In [7]:
```
sns.rugplot(normal_cell, label = 'normal', color = 'green', linewidth = 0.5)
sns.rugplot(diseased_cell, label = 'diseased', color='orange', linewidth = 0.5)
plt.legend()
```

Out[7]: <matplotlib.legend.Legend at 0x1a1bba8860>

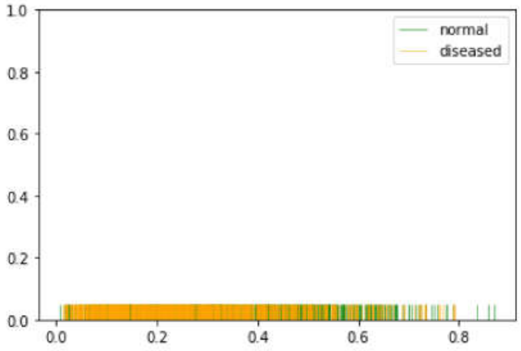

Implementing CSEA methods by calculating P_hit and P_miss and the Enrichment Score.

DESCRIBE P_hit, P_miss, ES

DESCRIBE P_hit, P_miss, ES

In [8]:
```
def ES_fast(score, s , p, interval):
    N = len(s)
    N_H = np.sum(s==1)
    m = 1/(N-N_H)
    power = score**p
    N_R = np.sum(power[s==1])
    h = power / N_R
    ES = [0]
    hit = 0
    miss = 0
    for i in np.arange(0, (len(power)-interval),interval):
        x = np.arange(i,i+interval,1)
        si = s[x]
        hit = hit + np.sum(h[x][si==1])
        miss = miss + m*np.sum(si==0)
        ES.append(hit-miss)
    return(ES)
```

CSEA for Diseased Cell Set

In [9]:
```
s = np.concatenate([np.repeat(0,len(normal_cell)),np.repeat(1,len(diseased_cell))])
score = np.concatenate([normal_cell, diseased_cell])
ranked = pd.DataFrame(np.asarray([s, score]).T,columns=['s','score'])
ranked = ranked.sample(frac=1)

ranked = ranked.sort_values(by='score',ascending=False)
s = np.asarray(ranked['s'])
score = np.asarray(ranked['score'])

ES = ES_fast(score,s,1,1)
plt.plot(np.arange(len(ES)),ES,'r-')
plt.title("Diseased Cell Set")
plt.xlabel("Rank in Ordered Dataset")
plt.ylabel("Enrichment Score (ES)")
plt.show()
```

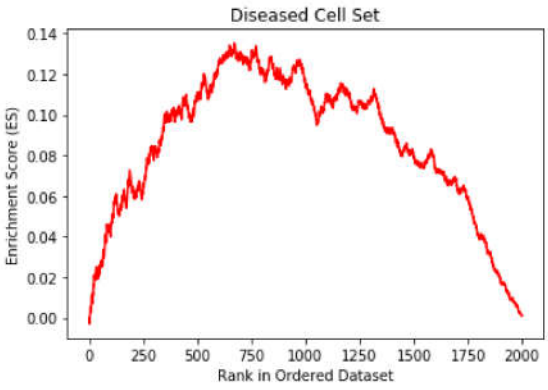

CSEA for Normal Cell Set

In [10]:
```
s = np.concatenate([np.repeat(1,len(normal_cell)),np.repeat(0,len(diseased_cell)) ])
score = np.concatenate([normal_cell, diseased_cell])
ranked = pd.DataFrame(np.asarray([s, score]).T,columns=['s','score'])
ranked = ranked.sample(frac=1)

ranked = ranked.sort_values(by='score',ascending=False)
s = np.asarray(ranked['s'])
score = np.asarray(ranked['score'])

ES = ES_fast(score,s,1,1)
plt.plot(np.arange(len(ES)),ES,'r-')
plt.title("Normal Cell Set")
plt.xlabel("Rank in Ordered Dataset")
plt.ylabel("Enrichment Score (ES)")
plt.show()
```

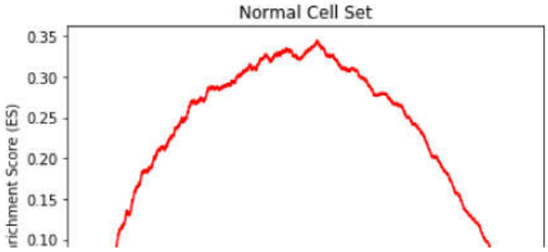

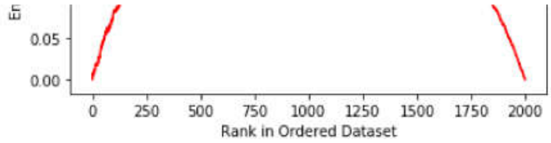

In [11]:
```
permuted=[]
n_permute = 1000
for i in range(n_permute): #randomizing the gene set signature or the labels
    perm = np.random.permutation(len(s))
    es = ES_fast(score, s[perm], 1,1)
    permuted.append(es)
null = np.asarray([np.max(x) for x in permuted])
pvalue = 1-np.mean(np.max(ES)>null)
print(pvalue)
```

```
0.0
```

In [12]:
```
def CSEA(genelist, genenames, norm_X, condition, filt,p=1,interval=50,use_Zscore=False,n_permute=1000):
    norm_X = norm_X[filt,:]
    condition = condition[filt]
    exprs = [np.asarray(norm_X[:,i]).ravel() for i,x in enumerate(genenames) if x in genelist]
    gene = [x for i,x in enumerate(genenames) if x in genelist]
    exprs = np.asarray(exprs)
    exprs = pd.DataFrame(exprs.T, columns=gene)
    if use_Zscore:
        exprs = (exprs-exprs.mean(axis=0))/exprs.std(axis=0)
    else:
        exprs = exprs/exprs.mean(axis=0)
    score = exprs.sum(axis=1)
    score = (score-np.min(score))/(np.max(score)-np.min(score)) #normalization
    ranked = pd.DataFrame(np.asarray([condition, score]).T,columns=['s','score'])
    ranked = ranked.sample(frac=1)
    ranked = ranked.sort_values(by='score',ascending=False)
    s = np.asarray(ranked['s'])
    score = np.asarray(ranked['score'])
    msES = ES_fast(score, s, p=p,interval=interval)

    permuted=[]
    for i in range(n_permute): #randomizing the gene set signature or the labels
        perm = np.random.permutation(len(s))
        es = ES_fast(score, s[perm], p=p,interval=interval)
        permuted.append(es)
    null = np.asarray([np.max(x) for x in permuted])
    pvalue = 1-np.mean(np.max(msES)>null)
    return msES, permuted, ranked, pvalue
```

Comparing to GSEA

In [13]:
```
def write_gmt(diseased_cell, normal_cell):
    diseased = ["Diseased" + str(i) for i in range(0, diseased_cell.shape[0])]
    diseased.insert(0, "Diseased")
    normal = ["Normal" + str(i) for i in range(0, normal_cell.shape[0])]
    normal.insert(0, "Normal")
    all_data = (np.vstack((diseased, normal)))
    np.savetxt('geneset.gmt', all_data, fmt="%s", delimiter="\t")
```

In [14]:
```
write_gmt(diseased_cell, normal_cell)
```

In [15]:
```
def write_rnk(diseased_cell, normal_cell):
    diseased = ["Diseased" + str(i) for i in range(0, diseased_cell.shape[0])]
    normal = ["Normal" + str(i) for i in range(0, normal_cell.shape[0])]
    names = np.hstack((diseased, normal))
    rank = np.hstack((diseased_cell, normal_cell))
    all_data = np.vstack((names, rank)).T
    np.savetxt('geneset.rnk', all_data, fmt="%s", delimiter="\t")
```

In [16]:
```
write_rnk(diseased_cell, normal_cell)
```

In [17]:
```
gsea_obj = gseapy.prerank(rnk='geneset.rnk', gene_sets='geneset.gmt', outdir='gseapy', min_size=15,
               max_size=1000, permutation_num=1000, weighted_score_type=1, ascending=False,
               figsize=(10,10), format='png')
```

In [18]:
```
img = plt.imread('gseapy/diseased.prerank.png')
plt.figure(figsize=(10, 10))
plt.imshow(img, interpolation='bilinear')
plt.xticks(color='w')
plt.yticks(color='w')
plt.show()
```

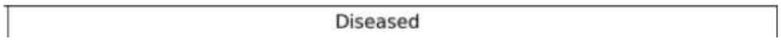

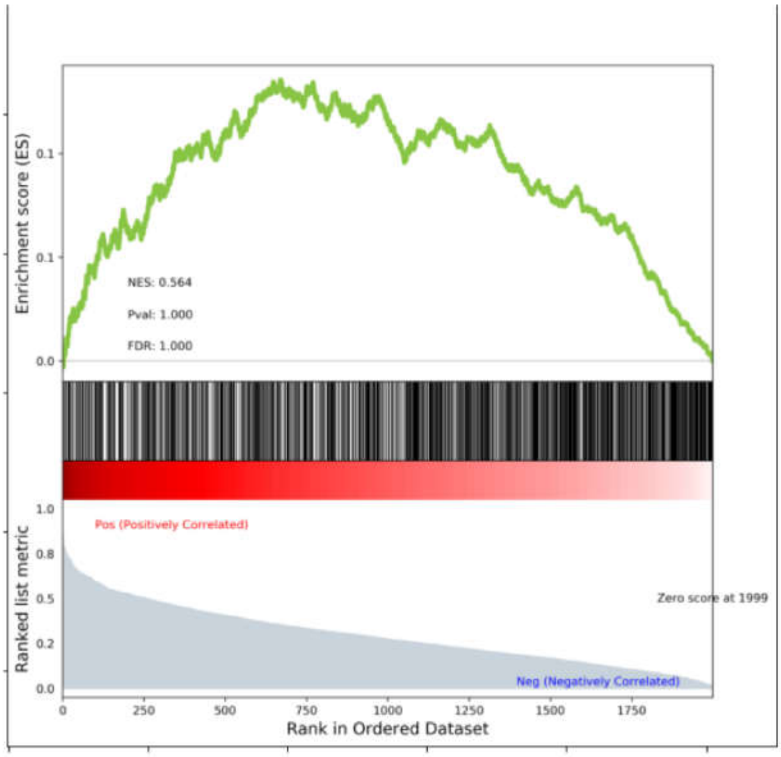

In [19]:

```
img = plt.imread('gseapy/normal.prerank.png')
plt.figure(figsize=(10, 10))
plt.imshow(img, interpolation='bilinear')
plt.xticks(color='w')
plt.yticks(color='w')
plt.show()
```

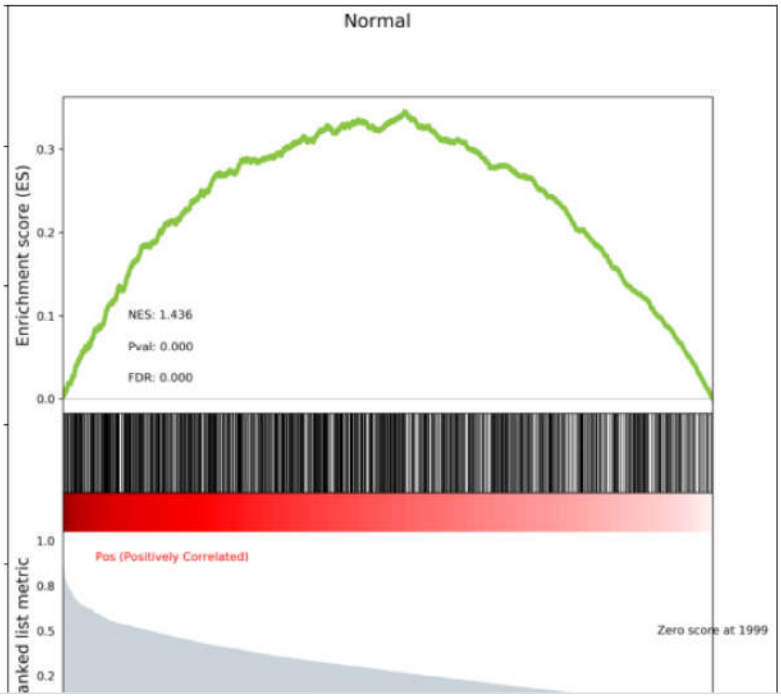

Supplement: Supplementary file 12 — Dataset 11 [file 41467_2019_14118_MOESM12_ESM.pdf]
