# Supplementary material for: Integrated single cell analysis of blood and cerebrospinal fluid leukocytes in multiple sclerosis
Source: Nat Commun. 2020 Jan 14;11:247. doi: 10.1038/s41467-019-14118-w (PMC6959356; doi:10.1038/s41467-019-14118-w)
Supplement: Supplementary file 13 — Reporting Summary [file 41467_2019_14118_MOESM13_ESM.pdf]

Corresponding author(s): Gerd Meyer zu Hörste

Last updated by author(s): Nov 5, 2019

Reporting Summary

Nature Research wishes to improve the reproducibility of the work that we publish. This form provides structure for consistency and transparency in reporting. For further information on Nature Research policies, see Authors & Referees and the Editorial Policy Checklist.

Statistics

For all statistical analyses, confirm that the following items are present in the figure legend, table legend, main text, or Methods section.

n/a	Confirmed	
☐	☒	The exact sample size (*n*) for each experimental group/condition, given as a discrete number and unit of measurement
☐	☒	A statement on whether measurements were taken from distinct samples or whether the same sample was measured repeatedly
☐	☒	The statistical test(s) used AND whether they are one- or two-sided<br>*Only common tests should be described solely by name; describe more complex techniques in the Methods section.*
☐	☒	A description of all covariates tested
☐	☒	A description of any assumptions or corrections, such as tests of normality and adjustment for multiple comparisons
☐	☒	A full description of the statistical parameters including central tendency (e.g. means) or other basic estimates (e.g. regression coefficient) AND variation (e.g. standard deviation) or associated estimates of uncertainty (e.g. confidence intervals)
☐	☒	For null hypothesis testing, the test statistic (e.g. *F*, *t*, *r*) with confidence intervals, effect sizes, degrees of freedom and *P* value noted<br>*Give P values as exact values whenever suitable.*
☐	☒	For Bayesian analysis, information on the choice of priors and Markov chain Monte Carlo settings
☐	☒	For hierarchical and complex designs, identification of the appropriate level for tests and full reporting of outcomes
☐	☒	Estimates of effect sizes (e.g. Cohen's *d*, Pearson's *r*), indicating how they were calculated

Our web collection on statistics for biologists contains articles on many of the points above.

Software and code

Policy information about availability of computer code

Data collection	FACS Diva (BD, https://www.bdbiosciences.com/en-us/instruments/research-instruments/research-software/flow-cytometry-acquisition/facsdiva-software), Kaluza (Beckman Coulter, https://www.beckman.de/flow-cytometry/software/kaluza)
Data analysis	FlowJo 10.4.1 (https://www.flowjo.com/solutions/flowjo), cellranger pipeline v2.0.2 (10X Genomics, https://support.10xgenomics.com/single-cell-gene-expression/software/pipelines/latest/what-is-cell-ranger), scVI (https://github.com/YosefLab/scVI), Scrublet (https://github.com/AllonKleinLab/scrublet), VISION (https://github.com/YosefLab/VISION), EdgeR (https://www.bioconductor.org/packages/release/bioc/html/edgeR.html), Bowtie2 (https://github.com/BenLangmead/bowtie2), FastQC (https://github.com/s-andrews/FastQC), Picard tools (https://github.com/broadinstitute/picard), SCONE (https://github.com/YosefLab/scone), gseapy v0.9.12 (https://github.com/zqfang/GSEApy), aod/betabin (https://github.com/cran/aod/blob/master/R/betabin.R), GraphPad Prism 5 (https://www.graphpad.com/), ImageJ v1.48 (https://imagej.nih.gov/ij/index.html), R 3.4.4 (https://www.r-project.org/), RStudio 1.1.447 (https://rstudio.com/products/rpackages/), Python 3.7.4 (https://www.python.org)

For manuscripts utilizing custom algorithms or software that are central to the research but not yet described in published literature, software must be made available to editors/reviewers. We strongly encourage code deposition in a community repository (e.g. GitHub). See the Nature Research guidelines for submitting code & software for further information.

Data

Policy information about availability of data

All manuscripts must include a data availability statement. This statement should provide the following information, where applicable:

- Accession codes, unique identifiers, or web links for publicly available datasets
- A list of figures that have associated raw data
- A description of any restrictions on data availability

Raw bulk and single cell RNA sequencing data from the present study have been deposited in GEO repository with the accession code GSE138266. The GEO can be accessed using the secure token cjktygoudfyrnij. We have also included this statement into the Data Availability Statement.
All processed, unmodified scRNA-seq data (differential expression data, GSEA and CSEA data) are included as Supplementary Dataset Tables. Technical scRNA-seq

information and data tables with details of the included patients are included as Supplementary Dataset tables. The source data underlying Figures 3F, 3G, 4B-F and Supplementary Figures 1A-C, 1E, 4C, 4D, 10B, 10D, 10F are provided in the Source Data file.

Field-specific reporting

Please select the one below that is the best fit for your research. If you are not sure, read the appropriate sections before making your selection.

☒ Life sciences ☐ Behavioural & social sciences ☐ Ecological, evolutionary & environmental sciences

For a reference copy of the document with all sections, see nature.com/documents/nr-reporting-summary-flat.pdf

Life sciences study design

All studies must disclose on these points even when the disclosure is negative.

Sample size	No sample size calculations were performed ahead. Sample size was determined to be adequate based on statistical testing of magnitude and consistency between groups.
Data exclusions	Human samples not fulfilling the diagnostic criteria were excluded from the study. For details please refer to the methods section of the manuscript. No animal samples were excluded from the study.
Replication	In the human patient-based part of the manuscript, first the unbiased single cell RNA-sequencing technique was applied to generate hypotheses. Then subsequently, core findings were replicated using multi-color flow cytometry staining for specific T cell populations. One core finding was then replicated in animal experiments. All animal experiments were successfully replicated. For details please refer to the methods section of the manuscript.
Randomization	Human samples were allocated into experimental groups based on the described diagnostic criteria. For details please refer to the methods section of the manuscript. Animals were allocated into experimental groups based on their genotype.
Blinding	The physicians recruiting patients into this study were not blinded towards diagnosis. Subsequent experimental approaches were performed in a pseudonymized fashion and laboratory personal was blinded towards diagnosis of patients. Data analysis of transcriptomics data was performed in an unblinded fashion. Animal experiments was performed under blinding. Genotype of mice was not known to experimentator during data aquisiton and was only revealed upon data analysis.

Reporting for specific materials, systems and methods

We require information from authors about some types of materials, experimental systems and methods used in many studies. Here, indicate whether each material, system or method listed is relevant to your study. If you are not sure if a list item applies to your research, read the appropriate section before selecting a response.

Materials & experimental systems

n/a	Involved in the study	
☐	☒	Antibodies
☒	☐	Eukaryotic cell lines
☒	☐	Palaeontology
☐	☒	Animals and other organisms
☐	☒	Human research participants
☐	☒	Clinical data

Methods

n/a	Involved in the study	
☒	☐	ChIP-seq
☐	☒	Flow cytometry
☒	☐	MRI-based neuroimaging

Antibodies

Antibodies used	anti-mouse flow cytometry antibodies: CD3 (17A2, Biolegend); CD4 (RM4-5 or GK1.5, Biolegend); CD8 (53-6.7, Biolegend); CD11b (M1/70, Biolegend); CD11c (N418, Biolegend); CD19 (6D5, Biolegend); CD45 (30-11F, Biolegend); CD45RA/B220 (RA3-6B2, Biolegend); Foxp3 (eBioscience, FJK-16s); Gr1 (RB6-8C5, Biolegend); IFN-y (Biolegend, XMG1.2); IL-17A (eBioscience, eBio17B7); NK1.1 (PK136, Biolegend);<br><br>anti-mouse histology antibodies: CD3 (clone CD3-12, BioRad); Mac3 (clone M3/84, BD); B220 (clone RA3-6B2, BD); B220 (clone RA3-6B2, BD); Ki67 (clone SP6, Thermo Scientific)<br><br>anti-human flow cytometry antibodies: CD3 (UCHT1, Beckman Coulter); CD4 (13B8.2, Beckman Coulter); CD8 (B9.11, Beckman Coulter); CD14 (RMO52, Beckman Coulter); CD16 (3G8, Beckman Coulter); CD19 (J3-119, Beckman Coulter); CD25 (B1.49.9, Beckman Coulter); CD27 (1A4CD27, Beckman Coulter); CD45 (J.33, Beckman Coulter); CD45RA (ALB11, Beckman Coulter); CD56 (N901, NCAM16.2, Beckman Coulter); CD127 (R34.34, Beckman Coulter); CD138 (B-A38, Beckman Coulter); HLA-DR (Immu-357, Beckman Coulter)

Validation | All antibodies used are commercially available and were validated by the manufacturer.

Animals and other organisms

Policy information about studies involving animals; ARRIVE guidelines recommended for reporting animal research

Laboratory animals	All animals used are on the C57BL/6 background. CD4Cre, 2D2tg, B6.129S(FVB)-Bcl6tm1.1Dent/J mice59 were purchased from Jackson laboratories. The CD4CreBcl6flox strain was maintained by breeding the Bcl6flox allele to homozygosity. Mice from both sexes were used at the age of 8-14 weeks.
Wild animals	The study did not involve wild animals.
Field-collected samples	The study did not involve field-collected samples.
Ethics oversight	The animal research protocols were approved and supervised by the responsible state authorities (State Agency for Nature, Environment and Consumer Protection North Rhine-Westphalia, LANUV NRW) under reference number 84-02.04.2015.A319 and were performed in accordance with local regulations from the Tierschutzbüro der Medizinischen Fakultät Münster.

Note that full information on the approval of the study protocol must also be provided in the manuscript.

Human research participants

Policy information about studies involving human research participants

Population characteristics	Patients fulfilling diagnostic criteria for multiple sclerosis as outlined in the methods section and control patients with idiopathic intracranial hypertension were recruited into this study. For details please refer to the methods section of the manuscript.
Recruitment	Participants were were recruited from patients being treated in inpatient or outpatient clinics of the Department of Neurology with Institute of Translational Neurology at the University Clinic Münster.
Ethics oversight	The study was performed in accordance with the declaration of Helsinki and approved by the local ethics committees of the Ärztekammer Westfalen-Lippe and Westfälische Wilhelms University, under reference number 2015-522-f-S

Note that full information on the approval of the study protocol must also be provided in the manuscript.

Clinical data

Policy information about clinical studies

All manuscripts should comply with the ICMJE guidelines for publication of clinical research and a completed CONSORT checklist must be included with all submissions.

Clinical trial registration	no clinical trial
Study protocol	no clinical trial
Data collection	no clinical trial
Outcomes	no clinical trial

Flow Cytometry

Plots

Confirm that:

- [x] The axis labels state the marker and fluorochrome used (e.g. CD4-FITC).
- [x] The axis scales are clearly visible. Include numbers along axes only for bottom left plot of group (a 'group' is an analysis of identical markers).
- [x] All plots are contour plots with outliers or pseudocolor plots.
- [x] A numerical value for number of cells or percentage (with statistics) is provided.

Methodology

Sample preparation	CSF samples were taken by lumbar puncture and blood by venipuncture. Samples were processed for Flow cytometry as described in the materials and methods section.
Instrument	BD FACS Aria III, Beckman Coulter Gallios, Beckman Coulter Navios
Software	FACS Diva v8, Kaluza , FlowJo v10.6.1

Cell population abundance	Purity of sorted samples was ~99% and was assessed by re-aquisition of sorted samples during establishment of the protocol.
Gating strategy	All samples were first plotted in a foward scatter vs. side scatter graph or CD45 vs. side scatter to gate on events corresponding to leukocytes. Then it was gated on single cells using forward scatter heigth vs. width and side scatter height vs. width. Afterwards, different marker combinations were used to identify cell populations as described in supplementary figures.

☒ Tick this box to confirm that a figure exemplifying the gating strategy is provided in the Supplementary Information.
